# Opening strategies in the game of go from feudalism to superhuman AI

**DOI:** 10.1017/ehs.2025.10016

**Published:** 2025-08-26

**Authors:** Bret Beheim

**Affiliations:** Department of Human Behavior, Ecology and Culture, Max Planck Institute for Evolutionary Anthropology, Leipzig, Saxony, Germany

**Keywords:** artificial intelligence, collective innovation, cultural evolution, information entropy, population structure

## Abstract

How does information infrastructure shape long-term cultural evolution? Using over four centuries of professional game records from the game of Go, this study explores how strategic dynamics in opening moves reflect historical shifts in the ‘infostructure’ of skilled Go players. Drawing from recent work on how population size, AI, and information technology affect cultural evolution and innovation dynamics, I analyze over 118,000 games using measures of cultural diversity, divergence, and player network composition. The results show distinct eras of collective innovation and homogenization, including an early 20th-century explosion of novel opening strategies, a Cold-War-era die-off, and a recent increase in evolutionary tempo with the arrival of the internet and superhuman AI programmes like AlphaGo. Player population size shows an inverse-U relationship with opening move diversity, and a recent decline in strategic diversity has accompanied a shift in the player network, from many small subgroups to a few large ones. Surprisingly, the influence of AI has produced only a modest, short-lived disruption in the distribution of opening moves, suggesting convergence between humans and AI and incremental rather than revolutionary cultural change.

## Graphical abstract

**Figure:**
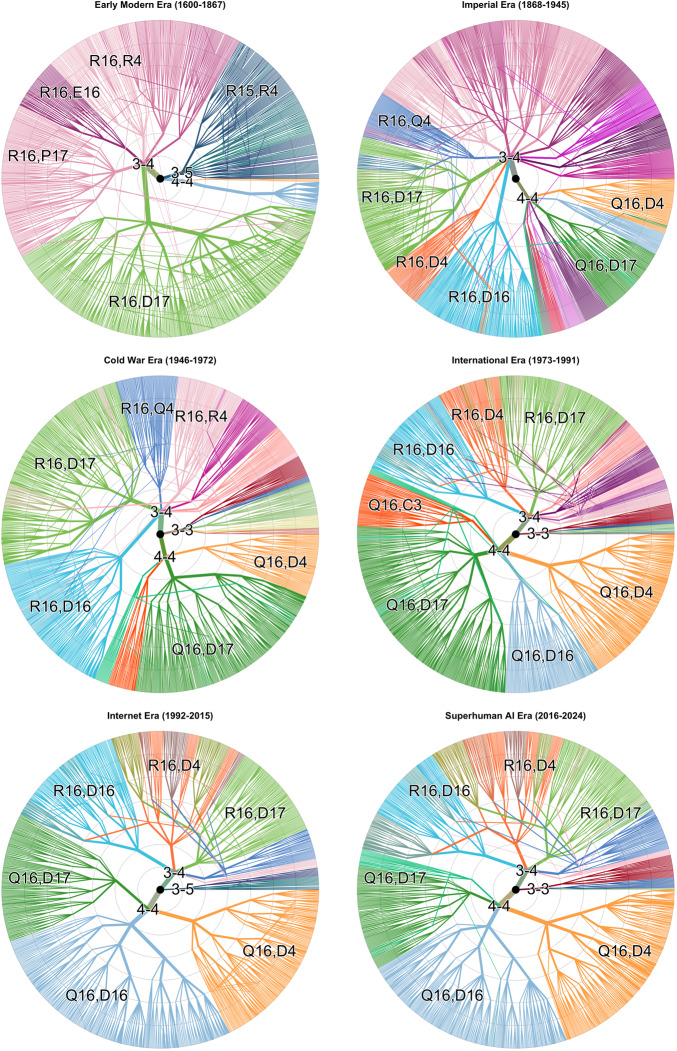

## Social Media Summary

Strategies in the ancient game of Go mirror deep societal shifts, from feudal traditions to today’s AI advisors.

## 1. Introduction

Learning from others can be effective when facing costly, difficult, or opaque decisions. This principle is an essential part of modern theories of cultural evolution (Cavalli-Sforza & Feldman, 1981, Boyd & Richerson, 1985, Mathew & Perreault, 2015, Kendal & Watson, 2023, Fogarty et al., 2024). But what happens if the quality and availability of that social information rapidly changes?

We are currently experiencing such a transition, towards AI-facilitated online lifestyles, which radically alters both the way information is shared and the nature of what is available to learners. How the rise of AI may alter the evolutionary dynamics of human culture has been the focus of several recent theory papers (Brinkmann et al., 2023, Farrell et al., 2025, Smaldino et al., 2025).

A common premise of this work is that the physical technologies and social institutions that structure information transmission, for example, the connectivity of a collaboration network, are likely just as important to human cultural evolution as our evolved cognitive mechanisms for social learning and decision-making. Sharper theory comes from good data and high-quality; however, long-term case studies are needed.

Here, I will explore such a dataset of four centuries of human strategic thinking in the game of Go. Made famous by the triumph of Google DeepMind’s AlphaGo programme in 2016 over the best human players, Go offers a rich historical record well suited to studying complex evolutionary dynamics. Empirical data can also sharpen our theories of cultural macroevolution. In particular, we can see how the rise and fall of different strategies within the game reflect larger shifts in the information infrastructure of Go, not least the recent arrival of superhuman artificial intelligence (SAI) agents such as AlphaGo. After a brief overview of the game itself, this introductory section will review recent theoretical and empirical work on this topic and pose a number of research questions that can be addressed using empirical game data.

### 1.1. Go as an observatory for studying long-term cultural evolution

The game of Go is ancient. Rulebooks describing the modern game date as far back as 557 CE, during China’s Northern Zhou dynasty (Shotwell, 2011, Zuyuan, 2011). Spreading from China, where it is known as *weiqi*, to Korea as *baduk*, and Japan as *go*, the ‘surrounding game’ today has active clubs and associations in cities and universities worldwide.

Like chess, Go is a board game played between two players, Black and White, who place stones on a large 19 × 19 square grid. The goal of the game is to enclose more of the board with your stones than your opponent, following specific rules for surrounding and capturing opponent pieces. Rather than the battles of attrition common in Chess, Go games tend to be relatively peaceful, and may play completely to the end with few stones taken as prisoners.

As a game of grand strategy, subtle influence, and long-term planning, Go is also famous for its computational complexity; within the first seven moves alone, there are over 
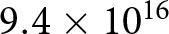
 (94 quadrillion) possible games. This open-endedness facilitates the long-term, cumulative evolution of ideas (Borg et al., 2022). A new opening strategy may do well and become a standard tool in players’ toolkits, or be discarded after an effective counter-strategy is developed, and theories of effective play have evolved considerably over the game’s history.

**Figure 1.**
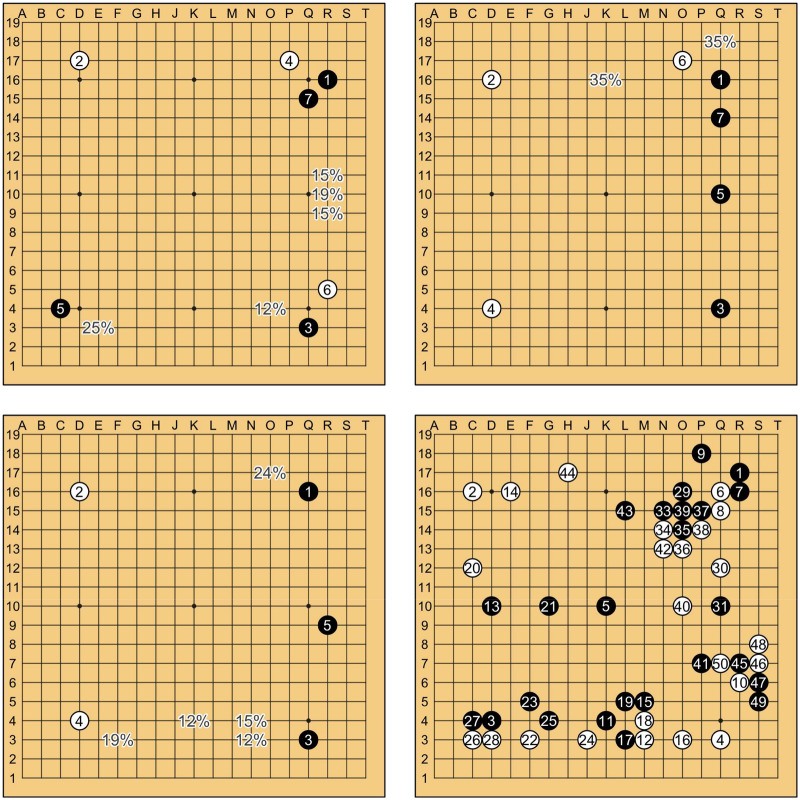
Four example openings in the GoGoD database, shown with the location and popularity of next moves by game percentages (less-common responses are not shown). *(top-left)* The first seven moves of the once-popular Shushaku opening sequence, which begins with Black 1 at the 3-4 point (Korschelt coordinate R16) followed by White’s 3-4 on the adjacent corner (D17). Such openings were popular in the 18th and 19th centuries but have since become extinct (Fig. 3). *(top-right)* The first seven moves of a sequence, which begins with Black 1 at the 4-4 point (Korschelt coordinate Q16). As described in the text, White’s potential response at K16 disappeared after criticism from a prestigious player. *(bottom-left)* The first five moves of the low Chinese opening, which also begins from the 4-4 point, with several standard variants for White 6. *(bottom-left)* The first 50 moves of a single game between Japanese professionals Go Seigen (as Black) and Honinbo Shusai (as White) played between October 1933 and January 1934. Here Black 1 is at the 3-3 point, Korschelt coordinate R17. Note that the standard Korschelt notation removes the ‘I’ from the column letters.

In complex, opaque systems like Go, classic social learning heuristics like conformist bias and prestige bias are likely to be used in identifying effective strategies. Consider this example: in 1996, the high-ranked professional player Go Seigen singled out the eighth move K16 (all moves in Korschelt notation) in a particular opening sequence shown in Figure 1, top right, as poor compared to other alternatives. Although K16 in this sequence had been increasingly popular in the late 1980s and early 1990s, with a respectable win rate, it vanished from professional play shortly after this verdict (Fairbairn & Hall, 2009).

This aligns with the basic logic of prestige bias in social decision-making (Jiménez & Mesoudi, 2019). If one of the strongest players of the 20th century, and the subject of the 2006 biographical film *The Go Master*, says a move is bad, they might know something that the typical player does not.

Similarly, a conformist bias can exploit the correlation between a particular strategy’s popularity and likely value to a social learner (Aplin et al., 2017, Youngblood, 2019). Consistent with this, in Go, there is evidence that players pick moves *because* they are popular (Beheim et al., 2014), after accounting for performance and past personal preferences.

The most famous event in Go’s history is the triumph of Google DeepMind’s AlphaGo over world champion Lee Sedol in 2016. In the years since, a variety of SAI programmes have become widely available, and under their tutelage human players have increased in both skill and experimentation of novel moves (Shin et al., 2023).

In all of Go’s long history, though, only a tiny fraction of possible paths in the game has been explored, by humans and now machines. Although Go is usually thought of as a competitive sport, we might more usefully think of it as a *collaborative* problem-solving task, one that has been running as a distributed, collective programme for almost two thousand years.

### 1.2. Exploring long-term cultural change in the light of infostructures

Smaldino et al. (2025) has recently emphasized the importance of the structural features of social and technological systems that enable and constrain the flow of information between individuals. They refer to these as ‘information architectures,’ and argue that these features are essential for understanding the evolutionary dynamics of cultural variants, perhaps more so than the operation of traditional cultural evolution mechanisms like social learning biases or frequency-dependent coordination effects.

For example, the evolution of information technology has been astonishingly rapid in the last several hundred years (Valverde, 2016). In the space of a few generations, we as a species have become hyper-connected by railroads, highways, jet airplanes, telephones, radio, television, cell phones, and the internet, and these sudden changes have had dramatic economic and social effects. The arrival of cell phones in developing economies has been shown to stabilize fishery prices almost immediately (Jensen, 2007) and buffer against economic shocks (Aker & Mbiti, 2010, Jack & Suri, 2014). Similar analyses have shown similar sudden impacts from the arrival of railroads in colonial India (Donaldson, 2018) and the printing press on the production of European science and art (Jara-Figueroa et al., 2019).

In applications to cultural dynamics, the concept of an information architecture is ripe, but the term suggests a system designed with a single coherent logic. This is true of CPUs or buildings, but not cities or societies. Describing a similar concept, Boyd and Richerson (1985) use the neutral phrase ‘cultural structures,’ though the concept is almost entirely underdeveloped in their seminal work. Preferring the analogy implied by ‘information infrastructure,’ I will (incautiously) use the term ‘infostructure’ to describe these partly evolved, partly designed systems, for the sake of brevity.

Generally, infostructures can be studied along three main dimensions: (1) the ways in which a particular configuration determines pathways *from* and *to* whom information can flow, (2) the *amount* of information that can be transmitted by a given pathway, and (3) the extent to which the *content* of that information is regulated, for example through the learning biases of the receivers or overt censorship or encryption by the senders.

The impact of these infostructural transitions on games like Go is probably dramatic. Although they are all playing essentially the same game, a student of Go in Nara in the year 1000 CE, Tokyo in 1890, San Diego in 1941, and Seoul in 2024 experience the game in radically different infostructures. As a result, we can predict that observed evolutionary dynamics of ideas within the game might map onto the kinds of social and technological affordances available to Go players.

In particular, we will focus on important shifts in the media of information dissemination, the size and social organization of player communities, the scope of interactions across great distances and times, and the availability of novel technology-based information sources such as game databases and SAI tutoring. With centuries of game data available, we can use the game of Go as a rich natural laboratory to study the ways in which humans process and act upon strategic information in different eras.

### 1.3. Population size, cultural diversity, and collective innovation

A key aspect of infostructure is the size and organisation of a population of cultural agents. As a basic pattern, cultural complexity tends to scale with the logarithm of population size (Bettencourt et al., 2007), implying cultural recombination acts multiplicatively in large populations (Arthur, 2009) and cultural loss in small ones (Henrich, 2004). Such scaling relationships have been found in a variety of domains, including Oceanic toolkit complexity (Kline & Boyd, 2010), rates of technological innovation, crime, and disease transmission (Bettencourt et al., 2007).

Not all measures of complexity have shown this pattern, however. Musical (Street et al., 2022) and linguistic (Wichmann & Holman, 2009) evolution are not clearly faster in larger populations, and the innovation literature has documented how large groups can become less innovative than small ones (Björk & Magnusson, 2009, Caldwell & Millen, 2010, Youngblood & Passmore, 2024). Similarly, economists report a slowing of progress across the applied sciences, as rates of innovation are not keeping pace with the growing number of scientists and the amount of research investment (Bloom, 2020).

Supporting these empirical findings, theoretical work has argued that population size matters less than how a population or metapopulation is *structured* (Baldini, 2013, Derex et al., 2018, Vidiella et al., 2022), such that a large but mostly unconnected social network can have a lower ‘effective’ population size than a small but moderately connected network (Deffner et al., 2022, Moser & Smaldino, 2023).

Further, the diffusion of beneficial innovations depends on the specific rules of interaction, for example, whether payoffs to individuals resemble producer scrounger dynamics or mutually beneficial division of labour (Beheim & Bell, 2024). In applied cases, certain institutions seem to encourage an over-reliance on copying (Youngblood & Passmore, 2024) and a dilution of expertise (Duran-Nebreda et al., 2022) even as population size grows.

The inherent potential within specific systems for additional cumulative improvements, such as how ‘open-ended’ the innovation space is (Borg et al., 2022), and the extent to which populations can become stuck within local optima (Winters, 2019), are also important mediating factors.

In Go, we can assess population size and structure by characteristics of the players, including their identity, nationality, and age, but also by the social relationships implied by the pair of players for each game. Go matches are functionally communication networks, as high-level players will usually review a game together after finishing it, exchanging thoughts on how well specific strategies performed. Knowing who played whom, and how often, creates a social network that reveals cliques, rivalries, cultural boundaries, and knowledge communities. Current theory indicates that such features may be useful for understanding the pattern and dynamics of behavioural diversity.

### 1.4. The role of SAI in cultural evolution

Today’s large language AI models are now able to produce photorealistic images of whatever we can imagine and provided detailed (and often overly confident) answers to any question. How these machine interactions might affect us is a central question of contemporary social science (Brinkmann et al., 2023).

Farrell et al. (2025) take the view that SAI are not intelligent agents per se, but rather a kind of social technology that allows ‘humans to take advantage of information other humans have accumulated,’ akin to an extremely sophisticated encyclopedia or online search engine. Bender et al. (2021) take a far more critical view of large language models as ‘stochastic parrots’ of their training data, which often reinforce conventional wisdom and amplify prejudices.

In Go, these points are likely true of programmes like the 2016 version of AlphaGo (which was trained on human games), but more recent SAI have developed their ‘understanding’ of the game without any human training data (Silver et al., 2017). In a series of recent papers, Minkyu Shin and colleagues argue that Go SAI are not just simply producing innovations that are blindly copied, but also helping to improve human reasoning (Shin et al., 2021) and encouraging human Go players to be more innovative (Shin et al., 2023). The dynamics of strategic evolution visible in historical Go game records offer a unique opportunity to study the impact of SAI on cultural evolution.

### 1.5. Plan for the analysis

With this conceptual framework, we can now examine how Go has changed in the *very long run*, starting from the early 1600s to the present day. I will begin this analysis by describing the historical game data in more detail and justifying specific target measures, for instance, the use of the first two moves of the opening and reasonable boundaries for different eras in the game’s long history. We can then ask how specific measures of cultural diversity and cultural disruption change through the centuries, and reflect Go’s evolving infostructure – both the material infrastructure (e.g. cities, transportation, and information technology), rule sets (e.g. player handicaps, scoring methods, timing rules), and the social institutions (e.g. exhibitions, tournaments, regular matches, discussion groups, game institutes, and chatrooms) through which ideas are mediated.

## 2. Methods

### 2.1. Getting game data

Traditionally, game records in Go are recorded diagrammatically as a sequence of decisions made on the 19 × 19 grid of the game board (Fig. 1). That is, the first move (by Black), second move (by White), and so forth appear as numbered locations on the 361 grid intersections of the board. Although a complete game record may contain hundreds of moves, this compact format has facilitated the preservation of the decisions of each player for hundreds, and in some cases, thousands, of years.

This study uses the GoGoD database, a curated collection of high-level games, both historical and contemporary, maintained by Go historians since 2001. GoGoD draws data from many major professional leagues across different countries, as well as high-level exhibitions and tournaments. Historical games have been painstakingly digitized by the GoGoD curators into the digital SGF format, recording the date, the players, match circumstances, full move sequences, and outcomes.

Game data was read into R using the kaya package (Beheim, 2025). A handful of duplicate or mis-specified games were removed, and game years and opening sequences were cleaned and prepared for analysis. Because of the rotational and reflectional symmetry of Go moves, all games were standardized so that the first move always appears in the top-right quadrant of the board. This novel data transformation allows direct exploration of the strategic dynamics within the game in unique and powerful ways.

Beginning with 124,534 games in the July 2024 database, I flagged and removed 4,368 handicap games (i.e. Black began with stones on the board to balance a difference in player skill), 775 games without a valid date, and 24 games, which were played before the year 1600. Although the influence of SAI players is an important part of this study, we will exclude the 51 SAI players and any games in which they participated, limiting the sample to human games. After removing 828 such games, we have an analysis sample of 118,348 games spanning the years 1600 to 2024.

The GoGoD database can be largely divided into two major periods, in 1945, the end of World War II. Games before this point are almost entirely from Japan, and in the earliest period only a handful of players and games were represented from each year. After 1945, the number of games, players, and nationalities grew exponentially, with games after 2007 accounting for over 50% of the total sample (SI Fig. A1). Certain calculations (e.g. Shannon move diversity) will be affected by such severe differences in sample size and must be controlled for. One way to characterize the structure of this population of players is with the games themselves, as two players coming together for a match reflects their shared cultural, linguistic, and national backgrounds, and these ties in aggregate describe a knowledge network. Statistics like the average path length, clustering coefficient (average transitivity) and a standard community detection algorithm (Clauset et al., 2004) will be used to formalize these properties of the match networks, and relate them to the cultural evolution of strategies.

Although the opening moves can be placed anywhere on the board, they tend to follow standard sequences known to both players, usually beginning with a Black move 1 at the 4-4 point (relative to one of the corners), the 3-4 point, or sometimes the 3-3 point (Beheim et al., 2014). Popular opening sequences stemming from these moves, along with a real game record, are shown in Fig. 1.

### 2.2. Characterizing different eras of social learning in Go

To represent major differences in the infostructures experienced by Go players across the centuries, I will demarcate six eras by major historical events relevant to the game.

#### 2.2.1. The Early Modern Era, 1600–1867

Historians refer to the Early Modern Era as the transition from agrarian feudalism to commercial, industrial societies in Europe and East Asia. In Japan, this encompasses the end of the Warring States period and the formation of the Tokugawa Shogunate in Edo (later named Tokyo), through to the opening of Japan to American trade in 1854 and the collapse of the shogunate in 1868. In China, this period includes both the Ming and Qing dynasties and in Korea, the Joseon period.

International contact between Go players was extremely rare, and the database during this period is divided into two separate clusters of Japanese and Chinese player networks. Chinese games during this period are not well documented in the database, besides the date and names of the players, and sometimes the location. Edo-period Japan had no organized professional leagues in the contemporary sense but did have several prominent Go schools with hereditary titles. Players from these schools competed in the famous ‘Castle Games’ played before the Tokugawa shoguns, which are some of the earliest games in the database (Shotwell, 2011).

Few people in subsistence agrarian societies could devote their lives to the game, and fewer still left game records that survived to the present day. Many games of this era are from prominent merchants, samurai lords, and in one case a Tokugawa shogun. Nevertheless, Early Modern players such as Honibo Shushaku had a notable impact on the opening theory (Figure 1, top-left). Within the GoGoD database, there are 2,930 games among 305 Chinese and Japanese players between 1600 and 1868.

#### 2.2.2. The Imperial Era, 1868–1945

The opening of Japan to American trade in 1854, and the subsequent Meiji Restoration, began a period of rapid Japanese industrialization. Copying the Prussian, American, and British models for industrial empire, Japan fought first Qing China and then Tsarist Russia in successive wars of expansion and occupied both Taiwan and Korea as imperial possessions.

As the industrial economy grew, more people became attracted to Go as a hobby, and the modern concept of professional players emerged during this time with the formation of the Japan Go Institute (*Nihon Ki-In*) in 1924. The imperial capital in Tokyo attracted highly talented players from throughout East Asia such as Chinese-born Wu Chuan (better known by his courtesy name, Go Seigen). High-level players competed for a number of prestigious titles in regular tournaments, with game records appearing in that week’s newspapers (Power, 1992).

Books on opening theory were numerous and began also appearing in translation, as Go became known to Europe and North America through East Asian immigrants and Western enthusiasts such as Oskar Korschelt and Arthur Smith.

In the 1920s and 1930s, Go players began experimenting with the rules of the game. Matches began implementing a default handicap that gives some number of points (called *komi* in Japanese) to the second player (White), who is at a slight structural disadvantage in sequential games. In subsequent eras, a *komi* of 4.5, 5.5, and eventually 6.5 points became standard (see SI Fig. A2). For this analysis, we can draw on 5,886 games played among 436 Japanese players between 1868 and 1945.

#### 2.2.3. The Cold War Era, 1946–1972

The events of the 1930s, 1940s, and 1950s had a profound impact on the nature of Go as a game. The Second Sino-Japanese War escalated into the Pacific War and eventually led to the unconditional surrender of Japan to the Allies and the dismantling of Japan’s overseas empire.

National reconstruction within the American sphere of influence led Japan to a historically rapid period of sustained economic growth and affluence. Mass electrification, highways, and high-speed bullet trains connected the country, while airlines made international travel routine. In Go, new connections between players could be formed via televised matches, a flourishing publishing industry, and growing international interest in the game in the United States and Europe (Power, 1992).

In China, the communist victory in 1949 led to the political separation of Taiwan and the mainland China, while Korea and Vietnam’s Cold War divisions likewise split each country into north and south. Once a diversion of the old aristocracy, Go became politically unpopular during China’s Cultural Revolution, and prominent Chinese players disappeared from the database in the 1960s.

South Korean Go became prominent during this time, with the Korean Baduk Association founded in 1945 by Cho Nam-ch’eol, who had studied in Japan in the 1930s. By the 1950s Korean tournaments had become common (Korea Baduk Association, 2008). During this era of political separation and tension, the database records 6,134 games played among 881 players, from Japan, China, and Korea.

#### 2.2.4. The International Era, 1973–1991

The end of the Cultural Revolution and Vietnam War, the normalization of ties between mainland China and the United States, and economic development throughout East Asia led to a sustained increase in interest in Go, and by the mid-1970s players had begun competing in regular international tournaments. Three distinct groups appear within the match network for China, Japan, and Korea, but the increased connectivity between players of this era is shown by a low average path length, with many between-country connections.

This era is also known for many new ideas in the game’s opening theory. Takemiya Masaki was an early adopter of the modern 4-4 as part of his ‘Cosmic’ style of non-defensive loose frameworks (Shotwell, 2011). Cho Chikun and Kobayashi Koichi popularized Q16,D4 openings in this era, and what are known in Japan and the West as ‘Chinese’ openings also became common (e.g. Figure 1, bottom-left). I refer to this period as the ‘International Era,’ with 1,972 players and 15,330 games, from Japan, South Korea, Taiwan, and mainland China.

#### 2.2.5. The Internet Era, 1992–2015

The arrival of online Go servers in the early 1990s allowed individuals, for the first time, to play and teach each other around the world in real time. Online play does not require the players live in physical proximity or speak the same language, massively expanding the size of player to networks. As the game continued to grow in popularity, it also became increasingly international, as top professional players appeared in Europe and the Americas. Today’s high-speed internet dramatically improved the bandwidth of these connections, allowing video tutoring, online courses, and livestreaming of matches.

The digital era also brought major advances in the study of the game. Large-scale digital archives of games, such as GoGoD, also came into being, and artificial intelligence research on Go was also greatly improved in online arenas like the Computer Go Server. Although many millions of online game records are available from this era, here, I will focus on 56,509 games among 3,334 human professional players (playing in-person rather than online) from around the world.

#### 2.2.6. The Superhuman AI Era, 2016–2024

To understand the impact of SAI on Go, it is important here to emphasize *how much better* these computer programmes are compared to humans. A standard metric for judging player skill is the Elo rating, which gives the relative chance of each player winning a match. While no human Go player has achieved an Elo higher than 3,900, Silver et al. (2017) report that AlphaGo Zero reached an extraordinary level of 5,185. Because Elo is relative, the only way such a number can be measured is to have SAI compete against other SAI – humans will just lose every match.

Far from ending the game’s popularity, though, SAI have been a boon to Go, as Lee Sedol’s defeat pushed the game into the popular consciousness and high-quality AI-powered learning resources became available. Since the triumph of AlphaGo in 2016, SAI programmes have become ubiquitous in serious study, serving as teaching and reviewing tools. Both amateur and professional Go players have noticeably improved with SAI tutors, and Shin et al. (2023) have found human games between 2018 and 2021 show a remarkable increase in opening innovation and an improvement in the quality of moves.

In the 2024 database, there are 31,559 games played by 1,852 human players in the SAI Era. All games in which one or both players were a SAI were removed, focusing this analysis exclusively on human play over time.

### 2.3. In Go, the first few moves structure the game

Go is famous for its vast complexity, and in studying the evolution of ideas in the game, there is an equally vast number of features to focus on. For example, a common way to learn theories of play is by proverbs such as ‘There is death in the *hane*’ or ‘Your opponent’s best move is your best move’ (Segoe, 1960). These imply recurring motifs that will appear in a variety of game circumstances, which could be treated as cultural traits.

Another approach is to track standard local sequences of play (called *joseki*), which may appear at different points in a game’s opening. Endgame strategies are particularly notable because perfect or optimal play can be determined exactly. Both because of its relative simplicity and because it has been a major area of focus by researchers over the centuries, we will here focus on the topic of opening theory within Go.

One way to conceptualize the space of explored openings is by sequence alignment algorithms, a technique for studying cultural diversity borrowed from genomics (Savage et al., 2022). Any two games can be represented as a series of moves and the similarity between the games as measured by the *edit distance*, the minimum number of substitutions, insertions, and deletions needed to transform one string of moves into another.

For example, consider these two opening sequences, played in 1916 and 2017, respectively:

Game 1: R16,D17,Q3,O16,Q14,D4,D15,C15,C14,C16,D14,E16,C10,K3,N3,R6,F3,R3,R4

Game 2: R16,D17,Q3,C3,D15,P16,P17,O17,Q17,C15,C14,C16,D14,E16,D10,D8,O16,Q5

Here board coordinates are printed in Korschelt notation, which indicates the row-column location of each move in the sequence (Fig 1). These two games begin similarly with R16,D17,Q3 but then diverge, only to resume with a popular followup sequence C15,C14,C16,D14,E16 at different points in their matches.

By Levenshtein edit distance, these game openings are relatively similar compared to most other games with completely different moves. For *N* games, we can assemble pairwise distances using the stringdist package (van der Loo, 2014) in R (v4.3.1), creating an *N* × *N* distance matrix. All calculations in here use Levenshtein distance (method lv), but are qualitatively equivalent to results using optimal string alignment (osa) or Damerau-Levenshtein edit distance (dl). Dimension reduction analysis is accomplished by classical multidimensional scaling (MDS), which transforms the distance matrix into a set of *N* points such that the distances between the points (in *k* dimensions) are approximately equal to the dissimilarities between games. This approach locates Go openings in a kind of *k*-dimensional map showing where, in an infinitely vast space of possibilities, humans tend to play.

**Figure 2.**
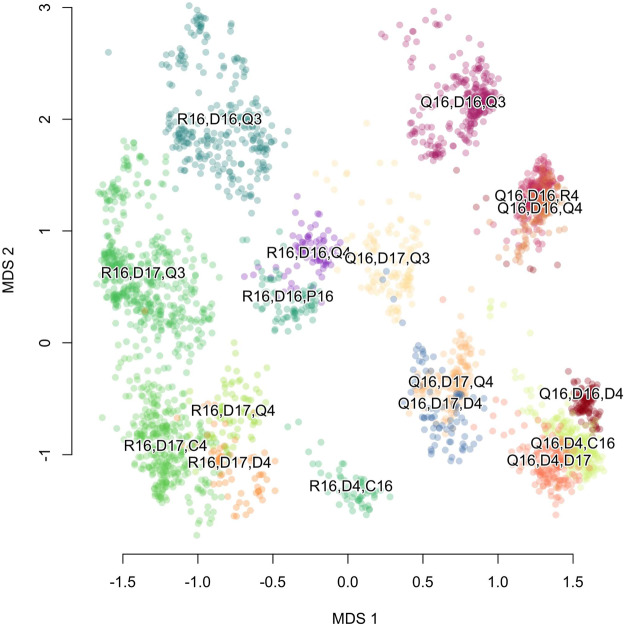
Multidimensional scaling (MDS) representation of 6,000 randomly chosen games (points) between 1600 and 2024, based on a matrix of Levenshtein distances of the first 50 game moves passed to R’s stats::cmdscale function. The x-axis (MDS 1) and y-axis (MDS 2) represent the two major dimensions of variation in openings, and correspond to the choice of the first move by Black and the second move response by White. Games that are more similar to each other are closer together, while points further apart have more divergent sequences within the first 50 moves. Distinct clusters (colours) correspond to specific sequences of the first three moves of each game.

Figure 2 shows the result of a two-dimensional MDS analysis of 6,000 randomly chosen games (points) between 1600 and 2024. We can see that opening sequences to a depth of 50 moves tend to cluster into groups along axes which correspond to the very first two moves: Black’s opening and White’s initial response. Games that begin with Q16,D16, for example, fall into the top-right corner of this latent space, while games that start with Shushaku-style R16,D17 openings appear in the bottom-left. Within these regions, the third move of the sequence tends to define discrete clusters, indicating a degree of overlap in subsequent moves in the game tree. Games with similar openings may completely diverge after the third move, for instance, R16,D17,C4 versus R16,D17,Q3, while others may have different openings and remain relatively similar to each other.

There are no inherent restrictions within the game that require these clusters to exist. Rather, out of the vast space of possibilities, human players have converged on a relatively small set of first and second opening moves, and by convention subsequent moves tend to be greatly influenced by these initial choices. Because of this useful fact, the two opening moves can become the basis for a cultural evolutionary analysis of the game.

### 2.4. Quantifying cultural diversity

#### 2.4.1. Balancing evenness and richness using Hill numbers

To understand the way various infostructures have affected strategic diversity, we need a principled way to characterize that diversity. The simplest, and crudest, representation is the number of unique variants or traits present, known in ecology as the trait “richness”.

Although it has uses, trait richness does not account for the relative abundance of each variant, captured in the concept of ‘evenness.’ For a given richness, evenness will be high if all variants are equally represented, and low if some variants are much more represented than others. In this way, evenness is a reciprocal quantity of the Gini coefficient of inequality familiar to the social sciences (Tran et al., 2021).

Today, scientific approaches to diversity usually begin with the framework laid out by Hill (1973), which combines both evenness and richness in a principled way. Hill diversity numbers can be calculated on a trait assemblage by

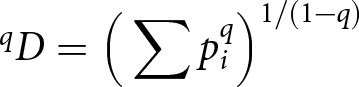


where *p_i_* is the frequency of each trait *i* and *q* is the ‘order’ of the diversity. Because of their generality, Hill numbers are an effective method of summarizing diversity within an assemblage, be it a set of species in an ecosystem or cultural products within a historical archive, for instance, recent analysis of survivorship bias of medieval European literature by Kestemont et al. (2022).

Order-zero diversity (*q* = 0 or ^0^*D*) is simply the richness of the assemblage, and as we increase *q*, we activate the importance of unequal weighting in the distribution. Order-two diversity (*q* = 2) is 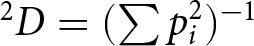, which we can recognize as a transformation of Simpson’s diversity index. Order-one diversity, which would be a compromise between pure richness and Simpson diversity, is undefined in Hill’s expression, but the limit as *q* → 1 exists and is

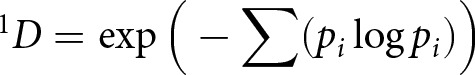


which is recognizable as the *exponentiation of Shannon information entropy*, 
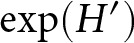
 (Jost, 2006).

As a measure of diversity, this statistic has a number of appealing features. Perfect evenness (equal representation) will return the number of variants in the assemblage (
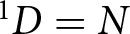
), and perfect unevenness means that a single variant dominates, so 
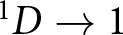
. Long-tailed, Zipf-like distributions common in linguistics and animal behaviour are effectively estimated using Shannon entropy (Kershenbaum et al., 2020). Information entropy specifically also appears to be a preference target in open-ended cultural systems, as artists seem to favour a ‘sweet spot’ between too much complexity and too little (Tran et al., 2021, McBride et al., 2025).

I will refer to 
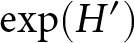
 as ‘Shannon diversity’ and take it to mean the *effective number* of variants within the assemblage. Taking board symmetries into account, among the 
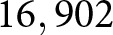
 possible options for the first two moves in Go, only 256 unique openings have been recorded in the database (the richness, ^0^*D*). The distribution of these moves is extremely skewed; the 8 most common variants cover 90% of database games, and only 49 openings cover 99% of games. Calculating the Shannon diversity on this distribution gives 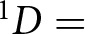 11.1 effective variants, equivalent to a population that played about 11 different moves with equal representation.

#### 2.4.2. Tracking changes in diversity

We can similarly use information theory to represent the *divergence* between two assemblages, or the same assemblage over time. The Jensen–Shannon divergence (JSD) measures the average Kullback-Leibler divergence (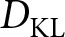) for two distributions *P* and *Q* from their averaged distribution *M*:

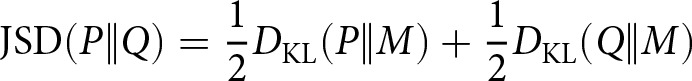


The JSD will equal 0 if two distributions are identical, and increases as they become dissimilar.

This information statistic is a principled method for studying evolutionary change, because it can account for the arrival of new variants, the disappearance of extinct variants, and the subtle changes between weightings of variants within a move distribution. The JSD is here calculated with the philentropy R package (Drost, 2014).

## 3. Analysis

Using concepts from information theory, evolutionary ecology and cultural evolution, we can examine the evolution of Go openings over the last four centuries. We can also connect these changes to major shifts in the infostructure within which players learn, and teach, the game. This includes important technological and geopolitical changes, but we will also examine the role of population size and community structure in maintaining strategic diversity in Go.

### 3.1. Tracking opening diversity through time

**Figure 3.**
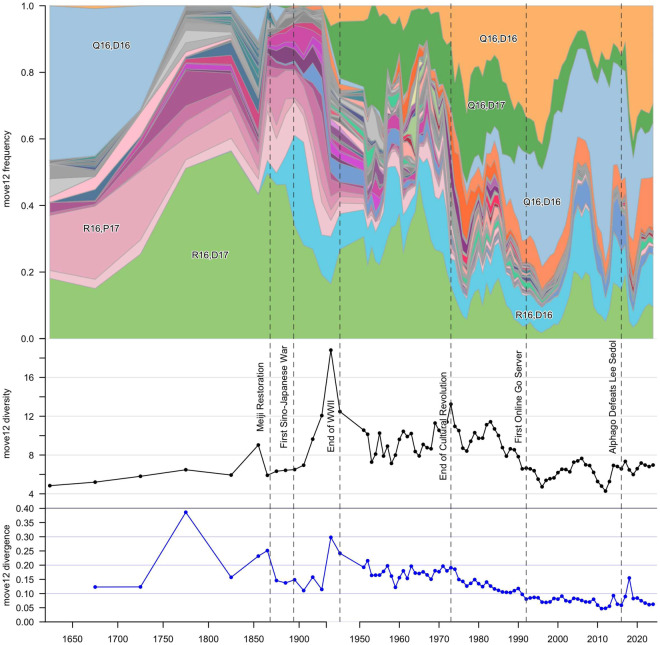
(*top*) Proportion of games by year for 256 opening variants (Black’s first move, and White’s response) in Korschelt coordinate notation over historical time (see key in Fig. 1). To account for differences in sample size in the database, games before 1950 were aggregated in 5-year bins, and games before 1850 were grouped in 25-year bins. Notable events signalling major changes to the infostructure of Go are marked. (*middle*) Shannon diversity (exp(*H*ʹ)) of the opening pair of moves during the same time period. To account for differences in sample size, 100 games were repeatedly drawn at random from each time period to calculate entropy, averaging over 100 bootstrap iterations. (*bottom*) Jensen–Shannon divergence calculated over the same bootstrapped sample as in the middle panel, comparing the current period’s opening move distribution with the previous period’s.

Drawing on the GoGoD database, Figure 3 illustrates the changes in opening strategies from the Early Modern Era to the present day, as represented by the identities of the first and second moves. To represent the higher-resolution, faster dynamics of the last century of play, Figure 3 changes the x-axis scale after World War II (SI Fig. A5 presents the same data on a single, fixed scale). For each point in time, we can also compute both the ^1^*D* Shannon diversity and the Jensen–Shannon divergence with openings of the previous time point. To account for large changes in sample size between time periods, both diversity and divergence calculations are averaged over 100 bootstrap samples.

Compared to those of Chess (McGrath et al., 2021, Lappo et al., 2023), the opening moves of the game of Go have undergone substantial turnover through time. The earliest games in the 17th century come from China, where stone placement for the first four moves was fixed at Q16,D4,D16,Q4. In Japan, where this rule had been abandoned, openings beginning with R16,P17 and R16,D17 were common for hundreds of years during the Early Modern Era, only to have virtually disappeared today. Variants such as Q16,D17 were discovered, came to prominence, and then mostly disappeared, while Q16,D4 vanished completely during the Edo period only to reemerge in the late 20th century with a new set of continuations.

Often, the dynamics of these changes correspond to important shifts in the infostructure of Go social learning. An enormous diversification of openings occurred in the Imperial Era, as Shushaku-style R16,D17 openings were abandoned in favour of radically novel strategies with unclear prospects (so-called hopeful monsters). Go historians refer to the early 1930s as the era of New Openings (*Shin fuseki*), pioneered by talented young professionals like Kitani Minoru and Go Seigen (Shotwell, 2011).

The postwar period saw the extinction of many of these experiments, and by the beginning of the Internet Era, nearly all *Shin fuseki* had vanished, except for a handful of enduring successes. A handicap favouring the second player, the *komi*, also became standard in the early 1950s (SI Fig. A2). As a result, the second player does not have to play as aggressively to overcome their disadvantage, while the first player cannot play as defensively as before.

Opening move diversity rose in the 1970s and 1980s, but the year-to-year information divergence declined, indicating incremental, rather than revolutionary, change. A notable shift occurred around 1985, in which Q16,D16 exploded in popularity and quickly displaced a variety of infrequently played openings. Go diversity during this time steadily decreased, reaching a low point first in 1996 and then again in 2012, the nadir of Go opening diversity.

The arrival of SAI in 2015 and 2016 coincided with a moderate increase in Go opening diversity, and a sharp spike in Jensen–Shannon information divergence in 2018. Subsequent years have shown this divergence to be temporary and modest; however, returning to pre-AlphaGo levels in the most recent years of high-level play. A similar spike, followed by a return to pre-AI levels, can also be observed in the JSD of game moves 3 to 5 (SI Fig. A3). This is a potentially meaningful result we will return to in the discussion.

Opening move dynamics within China and Korea are only fully visible in the International, Internet, and SAI Eras, but are largely consistent with the database-wide trends seen above (SI Figs. A6-A8).

### 3.2. Diversification of families of openings

**Figure 4.**
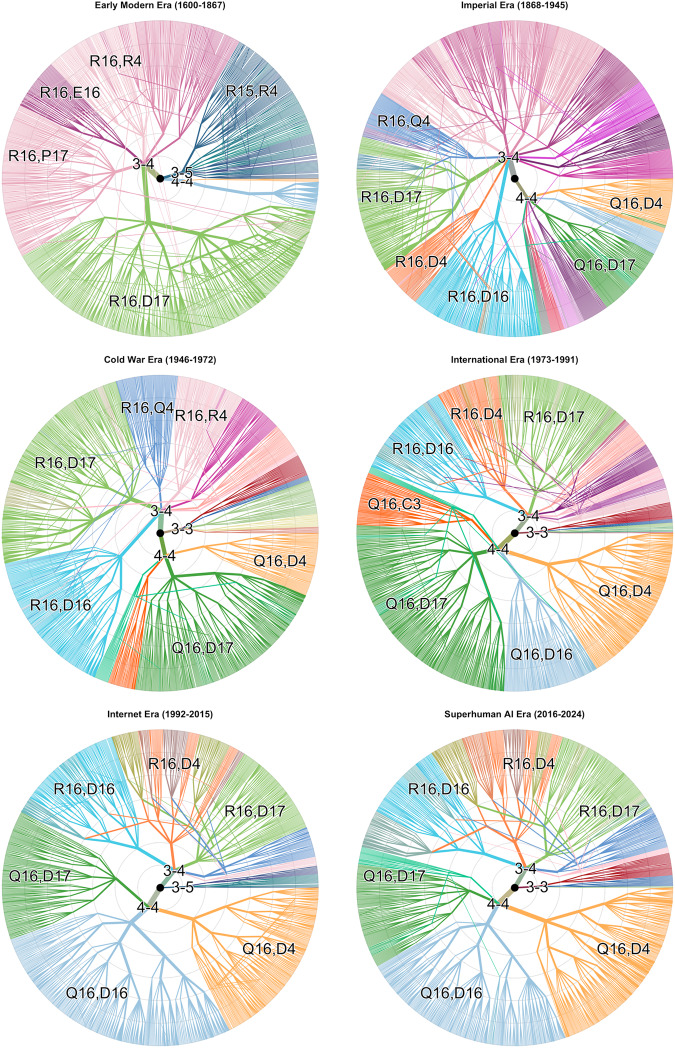
Decision trees showing common game sequences for the first seven moves. Beginning with the first move at the centre of the tree (black dot), each player sequentially chooses a point on the board to play their stone. Each branch’s colouration corresponds to a specific combination of first and second move, matching Fig. 3. Within the tree, cross-connections between branches indicate that two or more opening paths may lead to the same board state. The thickness of each line corresponds to the number of games of that era that followed this move sequence. For visual clarity, only the ceiling(exp(H_prime)) of the most common variants at each decision node are shown.

Although the first two moves are extremely effective at representing families of move sequences (Figure 2), they do not provide a complete picture of the diversification of follow-up moves within the opening trees. Figure 4 shows each opening as a *series of decisions* organized into Reingold–Tilford trees to a depth of seven moves. The popularity of each branch is represented by its line thickness. For visual clarity, only the most common branches from each decision node are shown (up to ceiling(exp(H_prime))).

Certain opening families, such as Q16,D4, maintain roughly the same number of continuations in recent history, while Q16,D16 experienced an extreme expansion during the Internet Era compared to the previous decades, only to contract in branch diversity in the era of SAI. More than half of the continuations during the Early Modern and the Imperial Eras are now extinct, though some such as R16,Q4 hang on as rare variants.

Although diversity increased with the arrival of the SAI, there was no major diversification of opening moves comparable to earlier eras. Several rare branches, such as Q16,C3 reappeared after vanishing during the Internet Era, but the major families of openings have remained remarkably stable since the mid-20th century.

### 3.3. Collective exploration of the strategic landscape of Go

**Figure 5.**
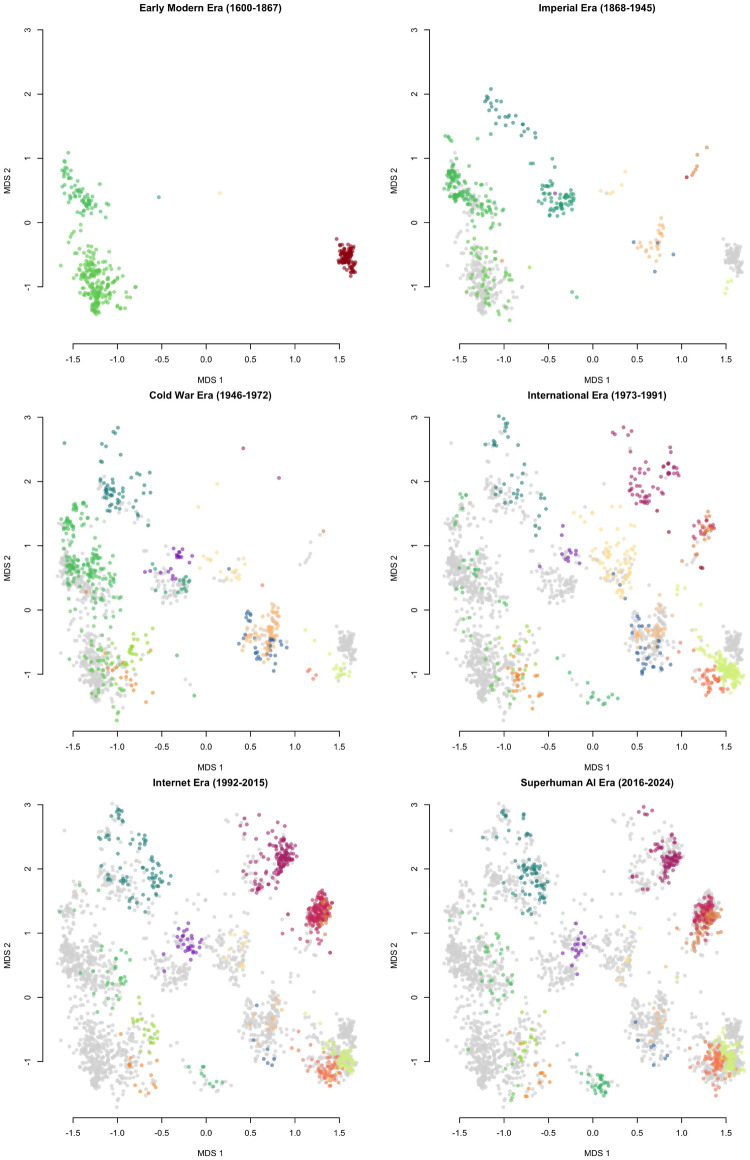
Exploration of the opening strategy space of Go between 1600 and 2024. Each panel shows the locations of individual games (coloured points) played in that era in latent MDS space described in Fig. 2. For each of the six eras after the first, the games of all previous eras are also displayed in grey.

The MDS map can also illustrate how Go players have explored the possibility space of openings over the last four hundred years (Figure 5) to a depth of 50 moves. For each era, we plot the location of 1,000 randomly chosen games (filled circles) and contrast the pattern of their locations with games of the previous era (open circles).

Early Modern Go openings were, relatively speaking, highly constrained to the vicinity of R16,D17 and Q16,D4 openings. The Imperial Era saw expeditions into several new opening spaces, with additional clusters appearing in the Cold War and International Eras. As new openings appeared, Go players researched how to develop them, accumulating various continuations as each cluster within the MDS map as it became larger.

The Internet Era saw the development and refinement of these ideas, especially exploration of the two Q16,D16 clusters, but no occupation of major new locations within the space. Rather, several explored locations, such as the bottom-left of the map, empty out during the Internet Era, and certain clusters become increasingly concentrated. This corresponds to the overall lower diversity of opening moves during this era (Fig. 3). In the years after the arrival of AlphaGo and other SAI, some small expansion of existing openings occurred, but – notably – no formations of new opening clusters.

### 3.4. The pace of opening evolution

Figure 3 also shows an apparent acceleration in the rate of evolutionary change in the last century, indicated by the need to change the scale of the *x*-axis. Contemporary oscillations between different opening paradigms (those beginning at the 3-4 point or the 4-4 point) seem to be getting faster and faster.

Following the methods used to study the rate of change in genetic and phenotypic evolution, we can quantify the speed of cultural evolution in this system by measuring the average magnitude (in standard deviations) of annual frequency changes for all variants at the first and second node of the decision tree through time.

**Figure 6.**
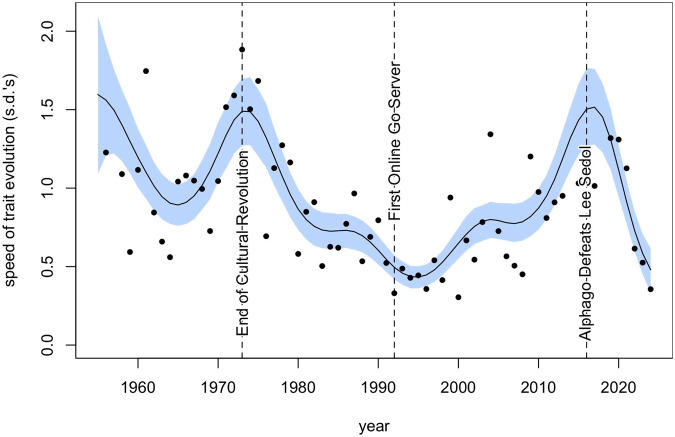
Magnitudes (in standard deviations) of the fluctuations in opening move frequencies since 1945, spanning the Cold War, International, Internet, and SAI Eras. Points are observed standard deviations in aggregate frequency changes each year. Shaded lines show means and 89% HPDI’s from a Gaussian process model fit using R’s cmdstanr package (Gabry et al., 2025).

Figure 6 indeed shows that the speed of strategic evolution has steadily increased since the arrival of the internet and experienced a brief peak immediately after AlphaGo’s victory. This is not a one-directional process, however, and in more recent years the pace of change has slowed.

### 3.5. Opening diversity and population structure

**Figure 7.**
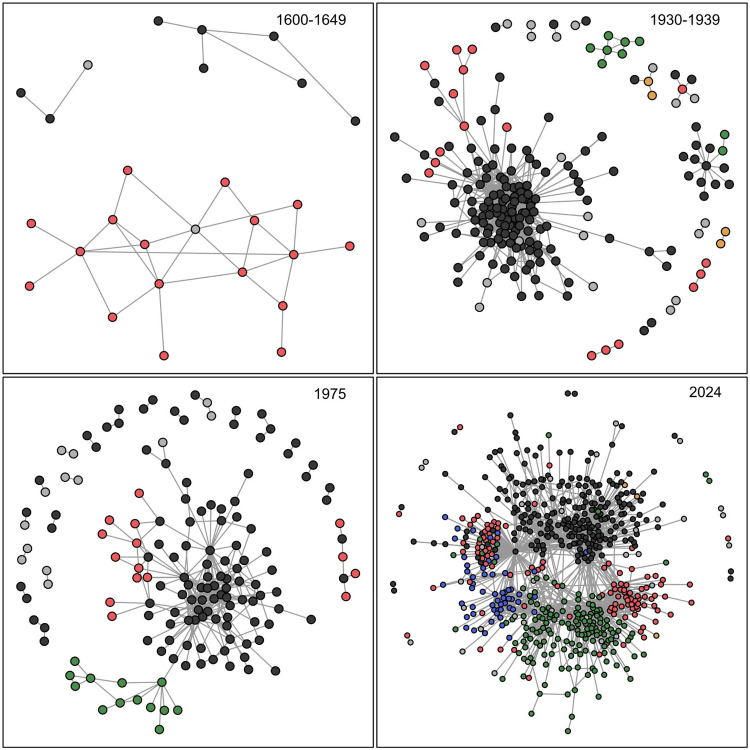
Network structure of Go players for the given time point or time period, weighted by the number of interactions (matches) seen in the database. When known, player nationality is given by node colouration (Chinese = red, Japanese = black, Taiwanese = blue, South Korean = green, European/American = yellow, unknown = grey).

The population of players at the beginning of the Early Modern Era was highly fragmented by feudal politics, language and cultural barriers, and long travel times. While the Imperial Era saw a rapid rise in the number of players, and their connectivity, the aftermath of World War II and the Cultural Revolution left the game unpopular in large parts of East Asia. Technological innovations, economic development, and the easing of international tensions during the International Era also increased the player base substantially, and today the international Go community is the largest it has ever been (Fig. 7).

**Figure 8.**
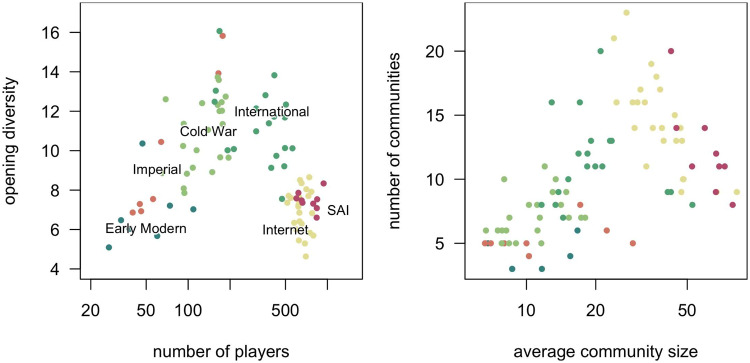
(*left*) ^1^*D* diversity of the first two moves and number of players for periods shown in Fig. 3. One extreme high-diversity point during the Imperial Era with 197 players and 
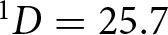
 moves is not shown. (*right*) Player network latent group size and count by community detection using igraph::cluster_fast_greedy on the main component of each time period’s match network (Csardi & Nepusz, 2006). In both panels, historical eras are indicated by point colours and labels.

Figure 8, left, isolates the relationship between player population size and opening diversity, finding an inverse-U relationship. The diversity peak in the early 1980s was associated with a player base about half the current size, and, interestingly enough, the largest populations also have the lowest levels of opening move diversity ever recorded.

Applying Clauset et al.’s 2004 community detection algorithm to the player match networks allows us to infer each player’s membership in latent social groups – cliques, tournament divisions, study groups, institutional affiliations, and so forth. The resulting communities and their average number of members can be counted through time. Mirroring the pattern of game diversity and log-population size, Figure 8, right, shows two distinct historical shifts: first, a flourishing of many relatively small communities of players, followed in more recent times by a small number of very large communities. This transition accompanies the observed drop in opening diversity.

## 4. Discussion

This analysis has described an ancient and ongoing process of collective innovation in Go opening theory, with periods of rapid diversification and relative stagnation. In concert with this, changes can be observed in the infostructure surrounding Go players – including information technology, social institutions, political and cultural boundaries, and social networks of play.

As the game became professionalized in the late 19th and early 20th centuries, new ideas were constantly innovated, refined, and abandoned through generations of players. The Imperial Era saw a complete transformation of opening theory and a tremendous rise in the diversity of openings, akin to the Cambrian Explosion of biological taxa 539 million years ago.

Extending the geological analogy, the immediate aftermath of World War II was accompanied by a mass die-off of opening branches and total abandonment of many long-standing moves, something like a cultural mass extinction, with an accompanying dramatic drop in diversity into the early 1950s. In the politically divided era that followed, those surviving branches in the decision tree, in turn, became increasingly developed as new variations appeared, filling out the main families of contemporary opening theory.

In the digital age, greater access to information, a larger and more connected player base, and the arrival of superhuman advisors likely drove an accelerating pace of innovation and counter-innovation in opening theory, culminating in a rapid burst of evolutionary change immediately after the appearance of AlphaGo and its successors.

Reviewing the evidence here, we can connect this story to a number of recent projects that emphasize the role of infostructure in cultural evolution: (1) the likely impact of the internet and now AI in the rate of evolutionary change, (2) the role of path-dependence in producing particular evolutionary outcomes, (3) the relationship between cultural diversity and both population size and community structure, and finally (4) the way in which Go and similar creative activities co-evolve with the perceptions and valuations of those activities.

### 4.1. The impact of SAI on opening theory is relatively small

Almost from the moment Lee Sedol was defeated, high-level Go players began discussing the possibility of SAI totally transforming the opening theory. Recent books by professional players have titles such as *How to Play Go the AI Way!* and *Fuseki Revolution: How AI Has Changed Go*, the latter of which recounts how, since AlphaGo, standard moves are disappearing and ‘their place being taken by new techniques invented by AI’ (Shibano, 2022). Seeing humanity defeated, data journalist and game theorist Oliver Roeder asked if the arrival of SAI really showed us that ‘humans just aren’t very good at Go to begin with?’ (Roeder, 2016).

It is true that many well-known opening sequences have been modified and improved upon by SAI. Learners today will often hear ‘the usual response at move X was Y, but an alternative move Z first suggested by AI is better’. This phenomenon is documented by Shin et al. (2023), who found a clear spike in novel variants within the opening tree in the years after AlphaGo’s arrival.

From a longer view, the burst of Jensen–Shannon divergence observed in 2018 was brief and historically modest. Although more people play Go than ever before, and the game has been rapidly evolving in the digital era, there has not been an explosion of new opening variations on the same scale as the Imperial Era, or a mass extinction of moves as in the tumultuous middle of the 20th century. Game openings since the Internet Era have been less strategically diverse than those of the 1960s and 1970s, when Go was rapidly becoming more international and ideas like Cosmic openings and variants of the ‘Chinese’ opening were under active development. Perhaps most significantly, the *families* of opening moves are not different post-AlphaGo compared to those established over the 20th century (Figure 4). In this way, the cultural evolution of contemporary Go shows incremental rather than revolutionary changes.

Taking these findings together with the observed acceleration of cultural evolution during the Internet and SAI Eras (Figure 6), we now see fast oscillations between a handful of regular opening sequences. This is categorically different from the slow replacement of a large variety of rare moves typical of Go’s earlier history; the basic evolutionary dynamics of the last 50 years are quite different from those of the previous 375. Whether this cyclic pattern will continue into the future is of course unclear, and a major innovation in opening theory may destabilize everything in the near future.

A similar disruption in chess three decades ago may prove instructive. After IBM’s DeepBlue beat Kasparov in 1996, there was a noticeable spike in human chess skill, especially among younger players in the early 2000s. Yet, although SAI training tools have become ubiquitous and players have been improving steadily since then, there have been no clear disruptive impacts from subsequent AI innovations like Stockfish or AlphaZero (the successor to AlphaGo Zero) (Bilalić et al., 2024). Similarly, SAI Era Chess openings seem to have changed only incrementally, rather than in large leaps (McGrath et al., 2021, Lappo et al., 2023). Taking this as a model, the recent decline in divergence in Figure 3 and SI Fig. A3 is possibly the end of the ‘SAI spike’ in Go evolution.

### 4.2. Contemporary openings are human–SAI convergent

Why some evolutionary processes are path-dependent and others are not is an outstanding mystery in cultural evolution. The number of Go strategies is effectively infinite, and much of the game’s evolutionary dynamics seem to depend on chance historical events; America’s opening of Japan in 1854, the chaos and destruction of the Imperial Era, the Chinese Cultural Revolution, the arrival of the internet, and the development of deep learning algorithms in the 2010s all had measurable impacts on Go evolution. Would the current diversity of strategies in the game be different had historical events unfolded differently?

Because modern SAIs trained themselves independent of human play, we can use them as a kind of natural experiment to answer this question. It was not at all obvious before AlphaGoZero whether SAI would rediscover human opening play while suggesting new innovations, or simply show us that we’d been doing it wrong the whole time. Contrary to Roeder’s hypothesis above, the results given here support the former scenario. A similar phenomenon occurred in Chess when AlphaZero bootstrapped its ‘understanding’ of the game and converged on many of the preferred human openings, for example, d4, e4, Nf3, c4, in roughly the same proportions (McGrath et al., 2021). Such examples of human–AI convergence speak to the efficacy of our cumulative, collective problem-solving abilities.

Still unanswered, though, is the question of why human cultural evolution settled on the major families of openings when it did, after such a long period of exploration. AlphaGo’s play was not necessarily revolutionary to players in 2016, but it would have been to players in 1916, and to players of the feudal era it would have been playing a totally alien game.

### 4.3. Group size and structure mediate cultural diversity

We can also see how the population structure of Go communities sustains behavioural diversity. Generally speaking, cultural diversity after World War II was highest when players were organized into many small communities. In the International, Internet, and SAI Eras, players assorted into fewer, larger groups as diversity in play styles declined. The historical eras with the largest number of opening variants also had an ‘intermediate’ number of players, relative to the feudal past and digital present.

This pattern is in accord with recent insights into cultural evolution of innovations. Networks that are completely disconnected prevent the spread of ideas altogether, but as people become more connected, moves can more quickly become extinct as the population too quickly converges on consensus or ‘canonical’ strategies. Partially fragmented, small world networks thus exist in a Goldilocks zone between overly connected and overly disconnected (Derex et al., 2018, Moser & Smaldino, 2023).

Besides population size itself, belief heterogeneity, metapopulation connectivity, communication noise, and institutional role specialization are all likely to influence the speed and nature of cumulative cultural evolution. As a unifying principle connecting all of these factors, Smaldino et al. (2025) have recently proposed the concept of ‘transient diversity’ to understand what features of a collective system will reliably cultivate new innovations.

### 4.4. Sabermetrics, aesthetics, and evolving perceptions of the game

The complex relationships seen here between strategic performance, move diversity and population structure are increasingly relevant, as many human creative and athletic pursuits become highly data-driven. As with Go, recent drops in diversity have been observed in shooting locations in professional basketball and batting strategies in baseball (Sheinin, 2025). Sabermetric analyses have led to an overall improvement in performance within each game, but at the cost of aesthetics and entertainment value.

In a broader historical perspective, the rise of AI-based and data analytic approaches to creative endeavours is one transition of many in how we perceive such activities. The game of Go has been a virtuous hobby for court aristocrats, a military training tool for samurai lords, a source of income for urban professional players, a computational challenge for artificial intelligence, and a sport to entertain fans. In the SAI Era, human preferences concerning elegance, excitement, or cognitive fatigue have been totally ignored in the advice of SAI advisors, whose only objective is to win. These transitions in meaning are difficult to quantify but equally worthy of study.

## Supporting information

Beheim supplementary material Beheim supplementary material

## Data Availability

The GoGoD database used here is commercially available at https://gogodonline.co.uk/. Analysis code and data to reproduce all figures and calculations are available at https://github.com/babeheim/go-learning-eras.

## Supplementary material

The supplementary material for this article can be found at https://doi.org/10.1017/ehs.2025.10016.
