# Supplementary material for: Opening strategies in the game of go from feudalism to superhuman AI
Source: Evol Hum Sci. 2025 Aug 26;7:e28. doi: 10.1017/ehs.2025.10016 (PMC12516593; doi:10.1017/ehs.2025.10016)
Supplement: Beheim supplementary material [file S2513843X25100169sup001.pdf]

SUPPLEMENTARY INFORMATION FOR "OPENING STRATEGIES IN THE GAME OF GO FROM FEUDALISM TO SUPERHUMAN AI"

Bret Alexander Beheim*[1]

[1]Department of Human Behaviour, Ecology and Culture, Max Planck Institute for Evolutionary Anthropology, Leipzig, Germany

1 Supplementary Figures

*bret_beheim@eva.mpg.de

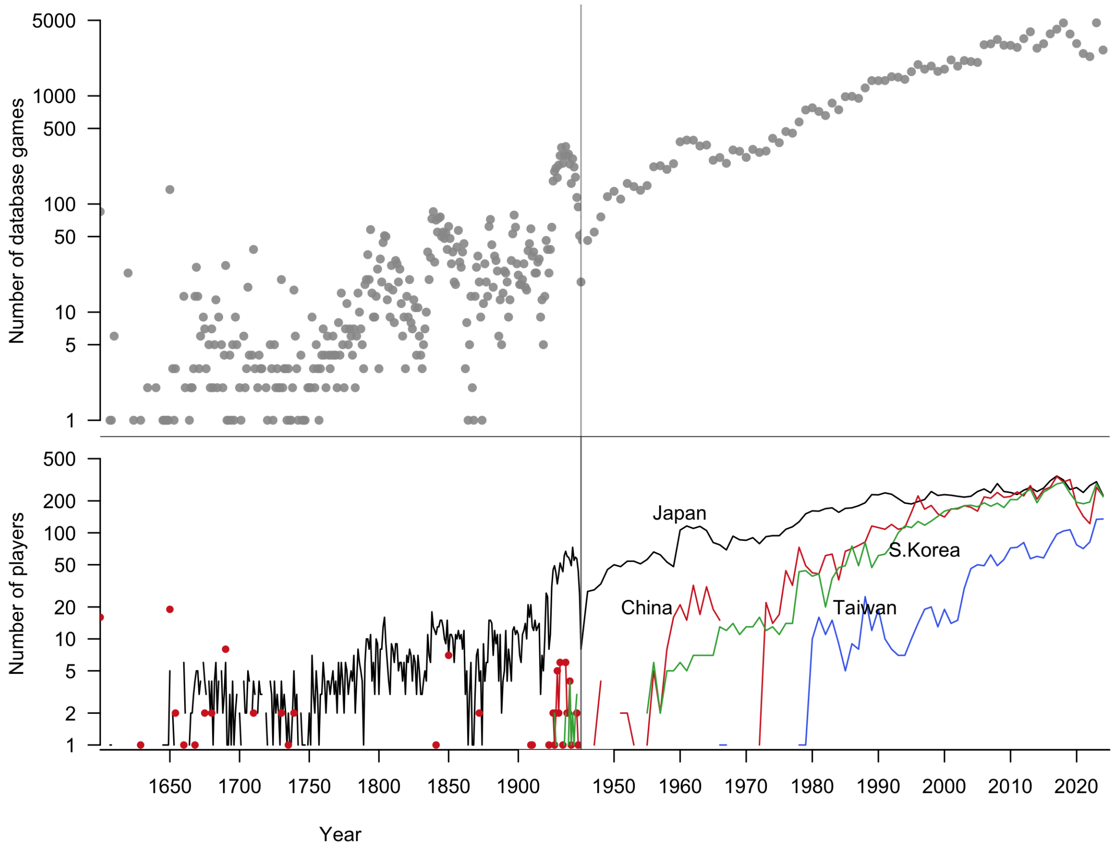

Figure A1: (*top*) Counts of 118,348 database games by year (note the logarithmic y-axis, and change of scale after 1945) (*bottom*) Counts of players by nationality and year, for 6,291 total players (note the logarithmic y-axis, and change of scale after 1945).

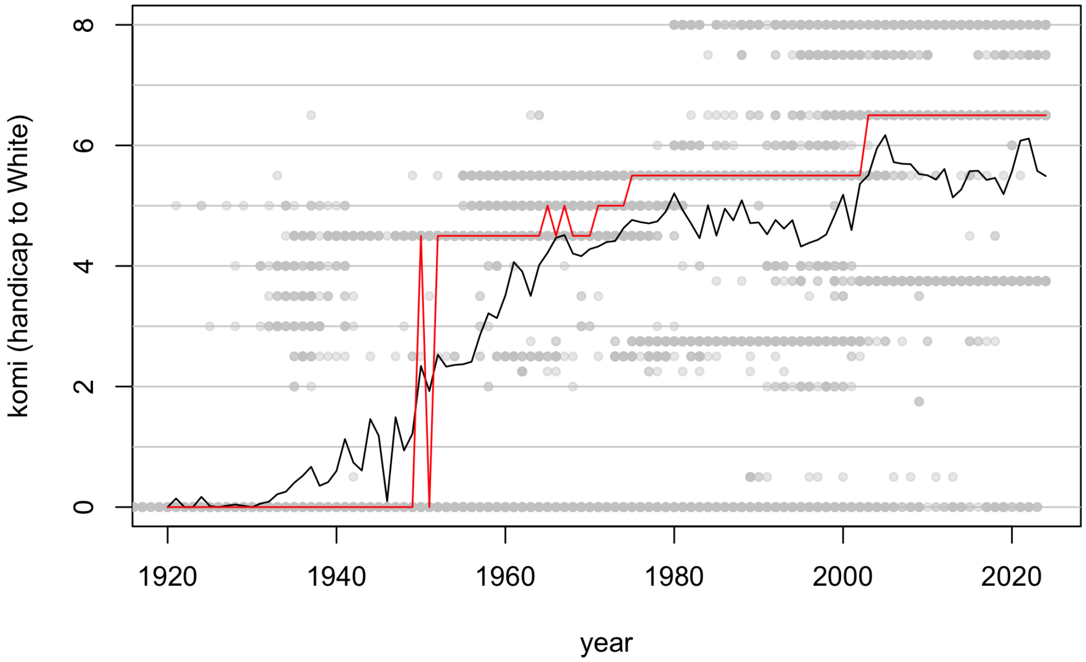

Figure A2: Number of handicap points awarded to the second player (a.k.a. *komi*) over the last century, showing individual games (gray points), the annual arithmetic mean (black line) and median (red line) across games.

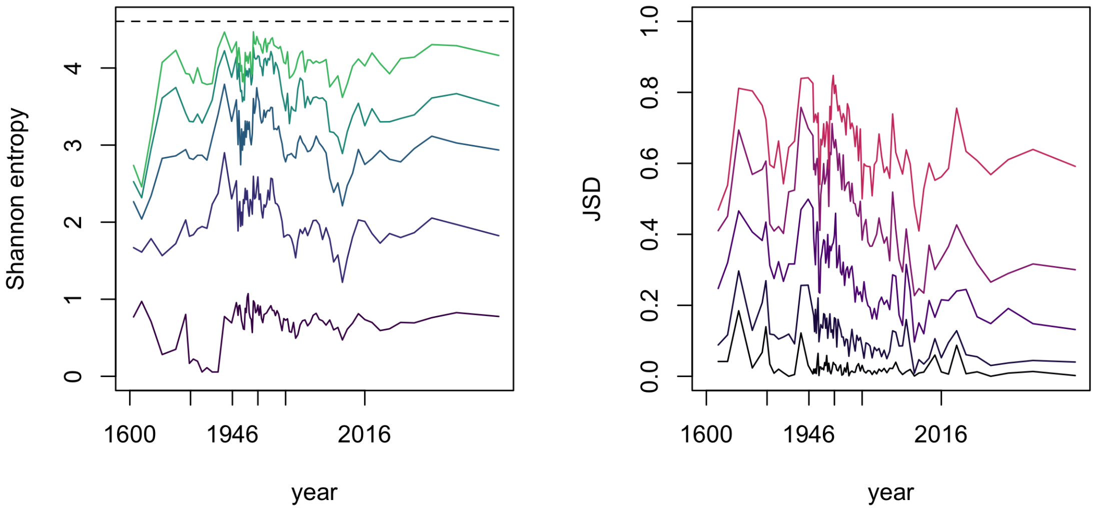

Figure A3: Diversity and divergence patterns in the first to seventh move across time.

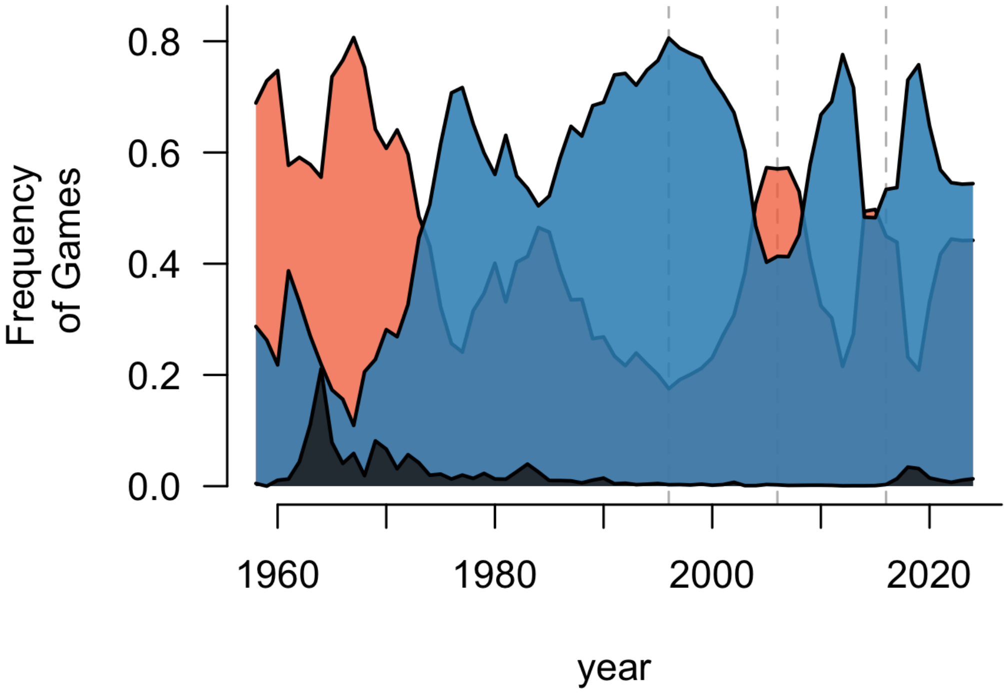

Figure A4: Frequencies of the three most popular first moves over time, showing the 4-4 opening (blue), the 3-4 opening (red) and the 3-3 (dark grey).

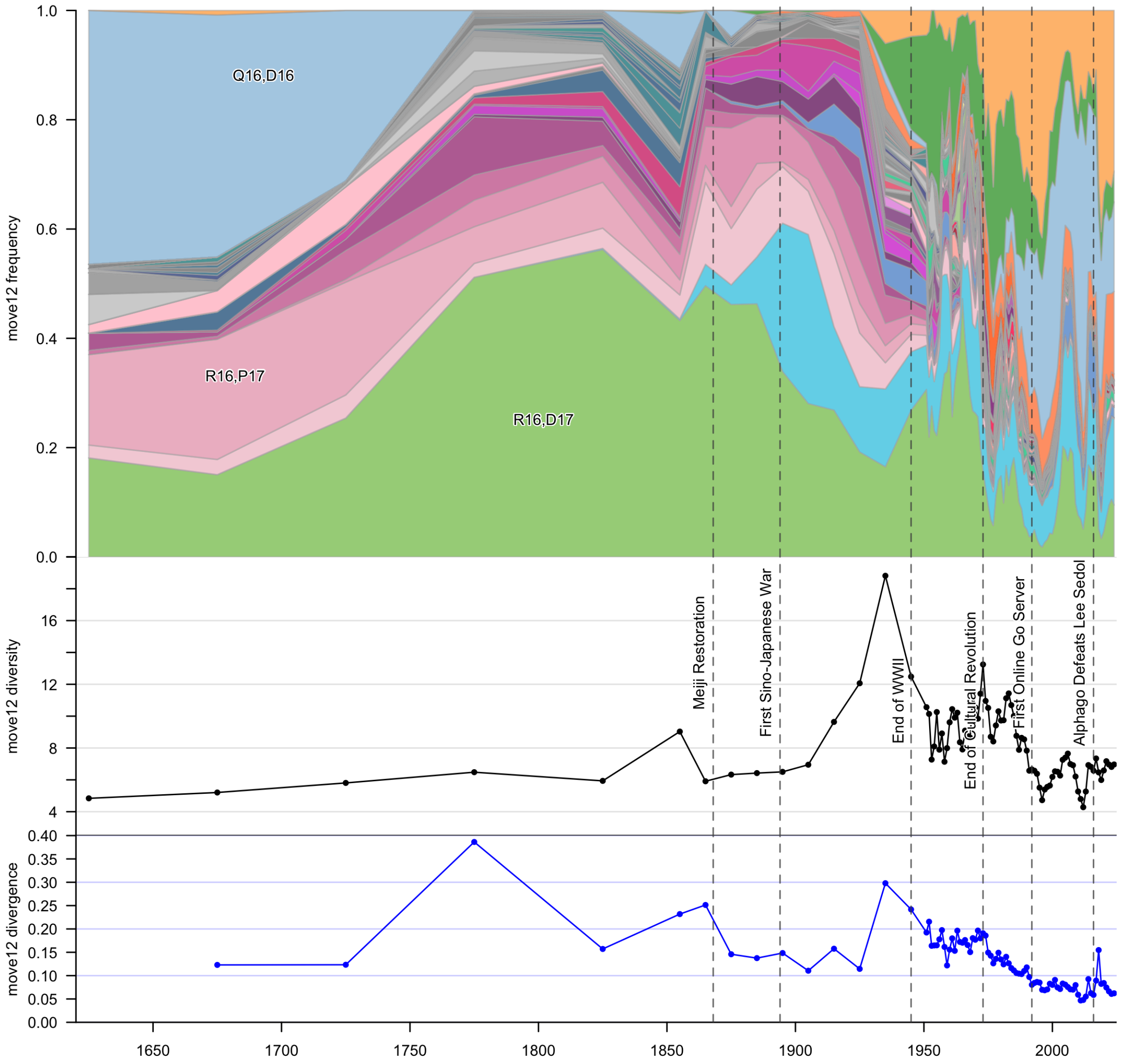

Figure A5: Proportion of games by year for 256 opening variants (Black's first move, and White's response) in SGF coordinate notation over historical time. To account for differences in sample size in the database, games before 1950 were aggregated in 5-year bins, and games before 1850 were grouped in 25-year bins. Notable events signaling major changes to the infostructure of Go are marked. (*middle*) Shannon diversity ($\exp(H')$) of the opening pair of moves during the same time period. To account for differences in sample size, 100 games were repeatedly drawn at random from each time period to calculate entropy, averaging over 100 bootstrap iterations. (*bottom*) Jensen-Shannon divergence calculated over the same bootstrapped sample as in the middle panel, comparing the current period's opening move distribution with the previous period's..

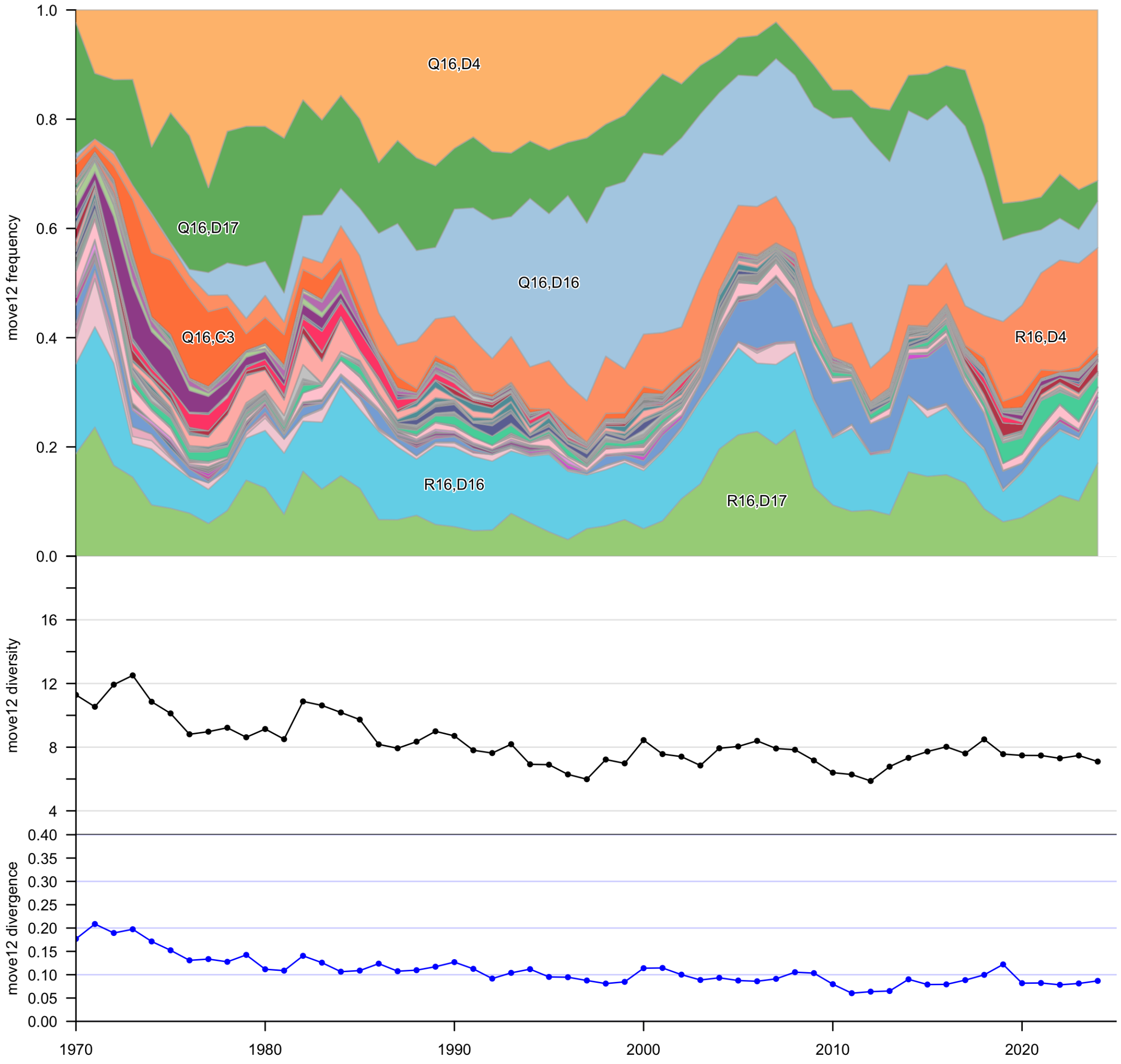

Figure A6: Proportion of games by year for 256 opening variants (Black's first move, and White's response) in over historical time, for Japanese players only. (*middle*) Shannon diversity ($\exp(H')$) of the opening pair of moves during the same time period. To account for differences in sample size, 100 games were repeatedly drawn at random from each time period to calculate entropy, averaging over 100 bootstrap iterations. (*bottom*) Jensen-Shannon divergence calculated over the same bootstrapped sample as in the middle panel, comparing the current period's opening move distribution with the previous period's.

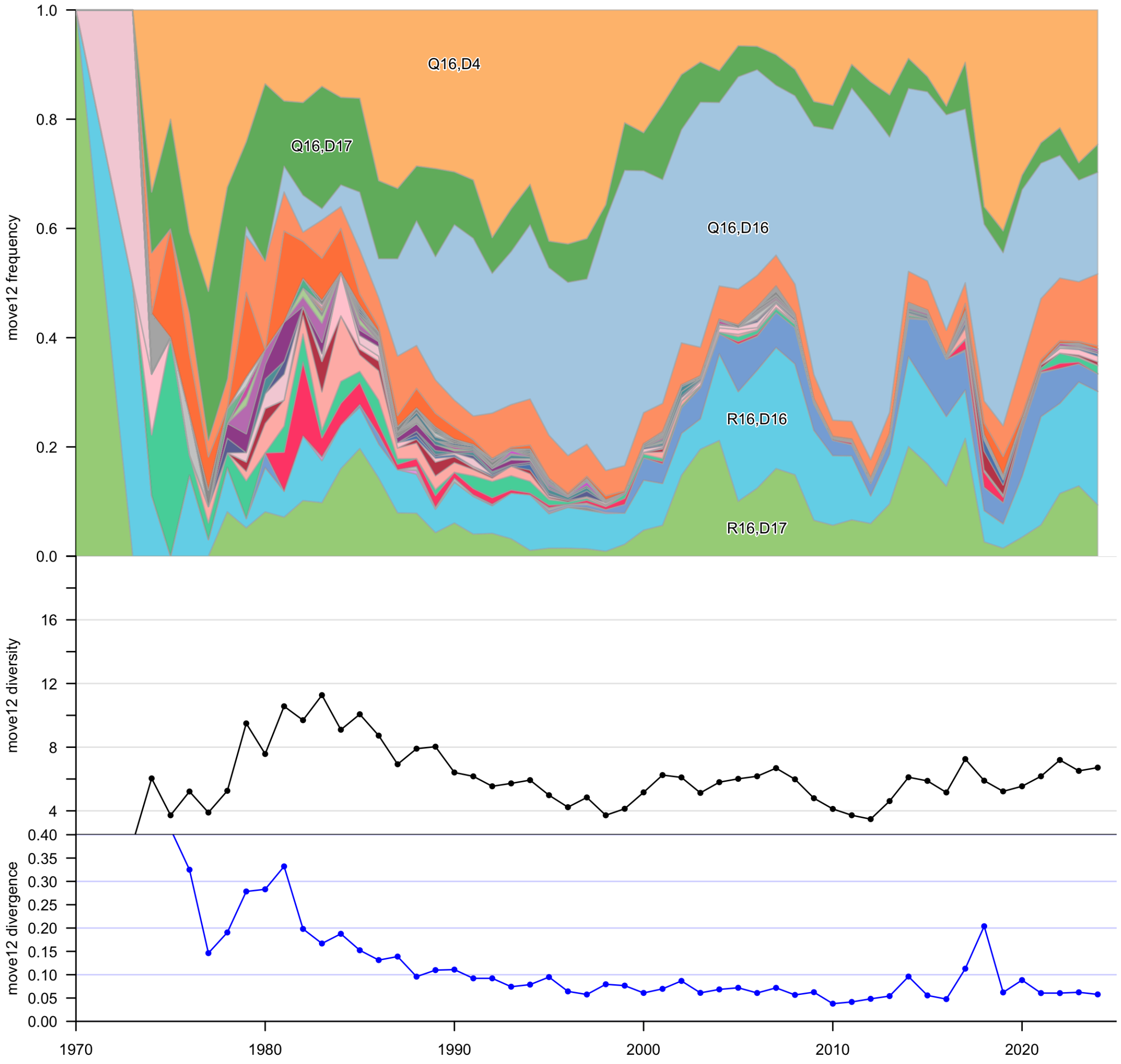

Figure A7: Proportion of games by year for 256 opening variants (Black's first move, and White's response) in over historical time, for Chinese players only. (*middle*) Shannon diversity ($\exp(H')$) of the opening pair of moves during the same time period. To account for differences in sample size, 100 games were repeatedly drawn at random from each time period to calculate entropy, averaging over 100 bootstrap iterations. (*bottom*) Jensen-Shannon divergence calculated over the same bootstrapped sample as in the middle panel, comparing the current period's opening move distribution with the previous period's.

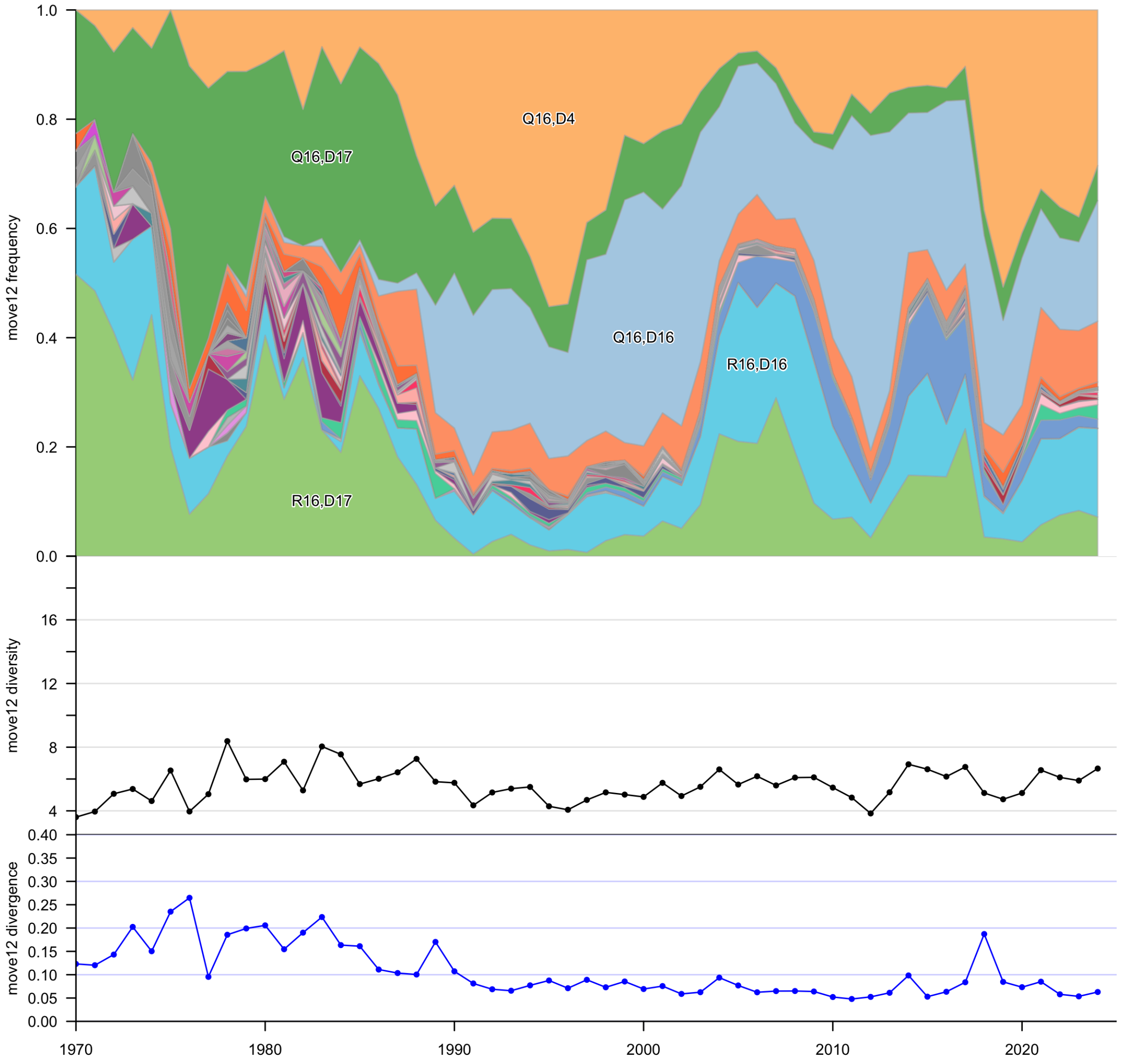

Figure A8: Proportion of games by year for 256 opening variants (Black's first move, and White's response) in over historical time, for South Korean players only. (*middle*) Shannon diversity ($\exp(H')$) of the opening pair of moves during the same time period. To account for differences in sample size, 100 games were repeatedly drawn at random from each time period to calculate entropy, averaging over 100 bootstrap iterations. (*bottom*) Jensen-Shannon divergence calculated over the same bootstrapped sample as in the middle panel, comparing the current period's opening move distribution with the previous period's.

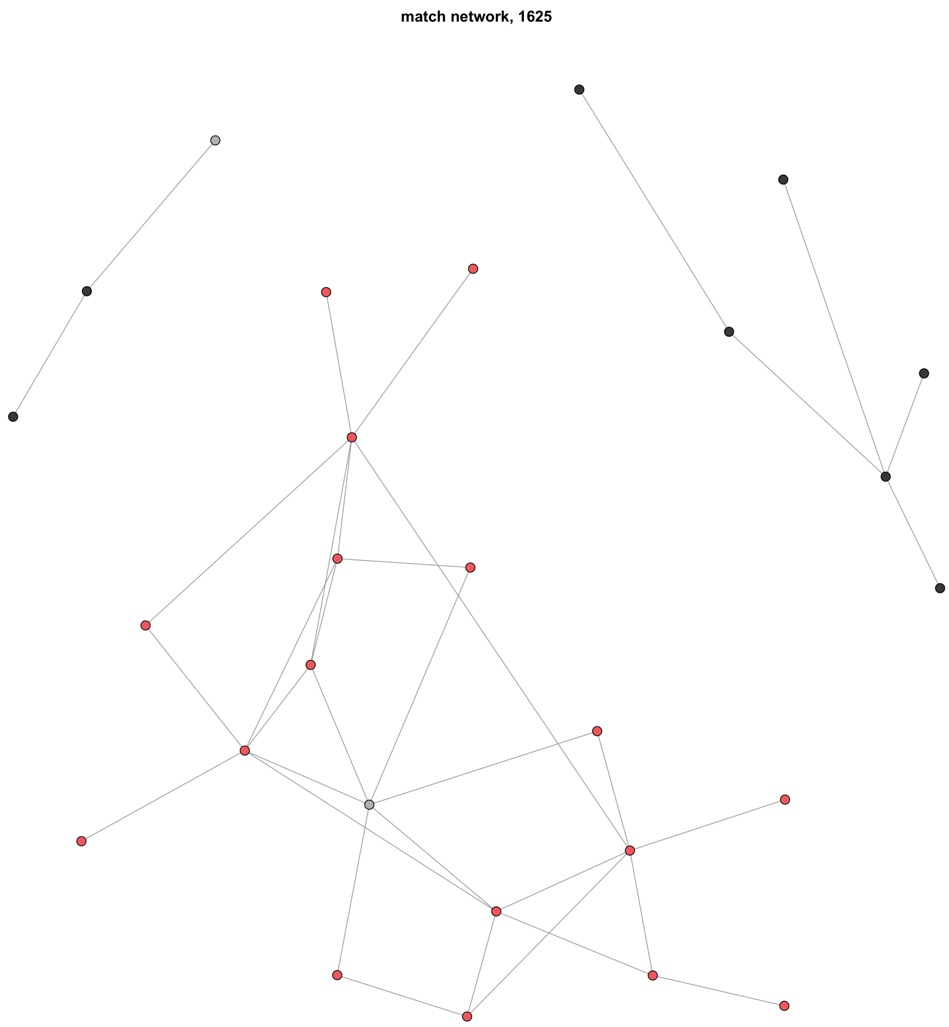

Figure A9: Connections between Go players (points) based on the number of games they played together in the year 1625, organized by a Fruchterman-Reingold layout. Node colors indicate player nationality (Black = Japan, Red = China, Green = South Korea, Blue = Taiwan).

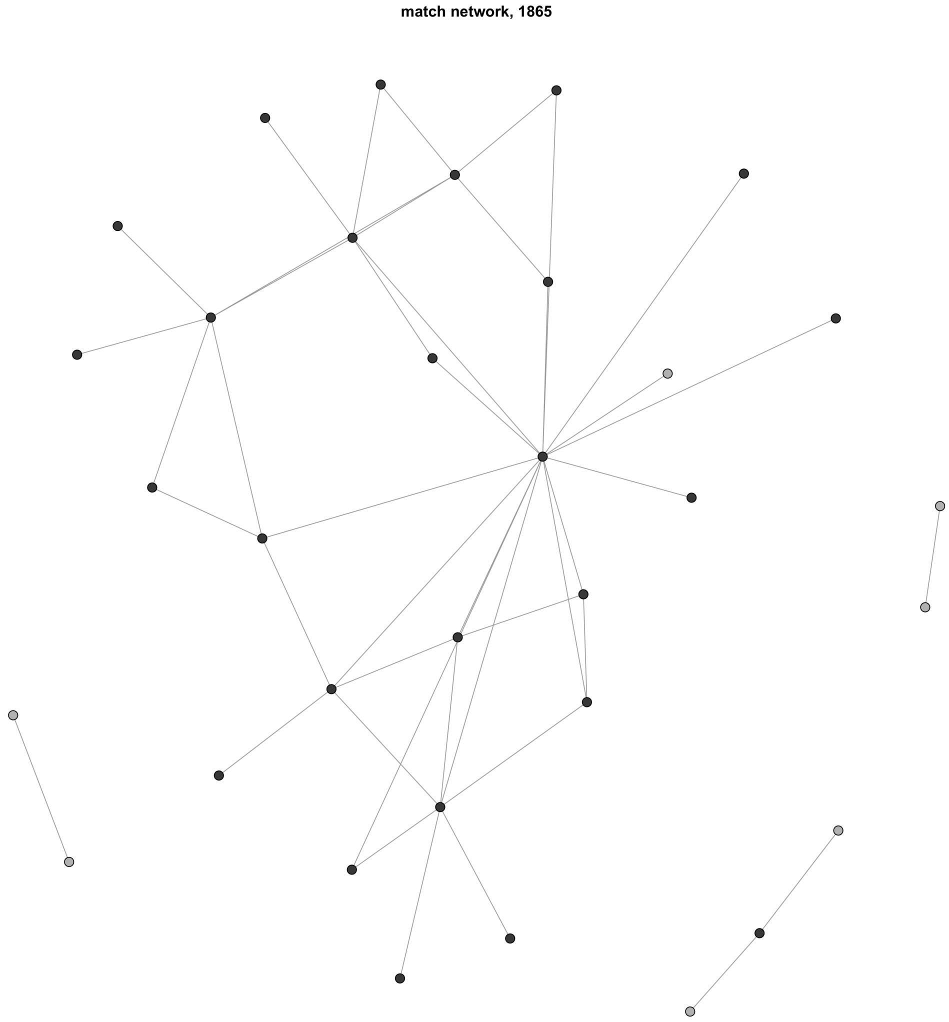

Figure A10: Connections between Go players (points) based on the number of games they played together in the year 1865, organized by a Fruchterman-Reingold layout. Node colors indicate player nationality (Black = Japan, Red = China, Green = South Korea, Blue = Taiwan).

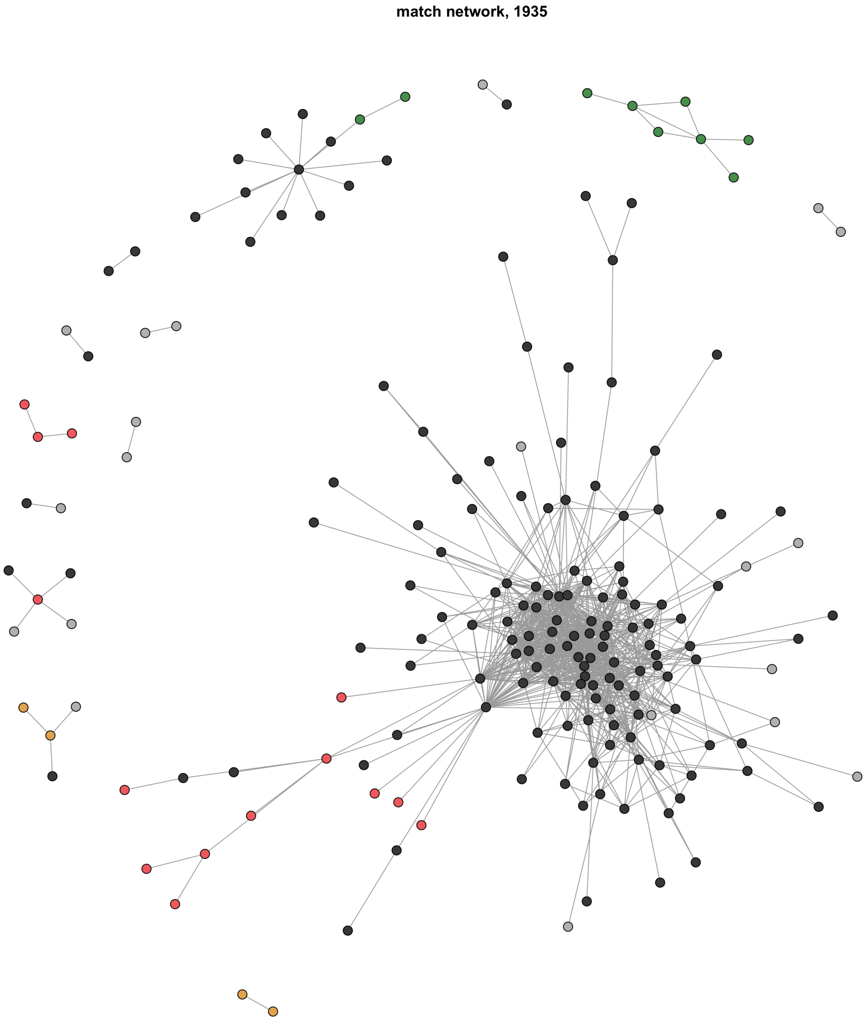

Figure A11: Connections between Go players (points) based on the number of games they played together in the year 1935, organized by a Fruchterman-Reingold layout. Node colors indicate player nationality (Black = Japan, Red = China, Green = South Korea, Blue = Taiwan).

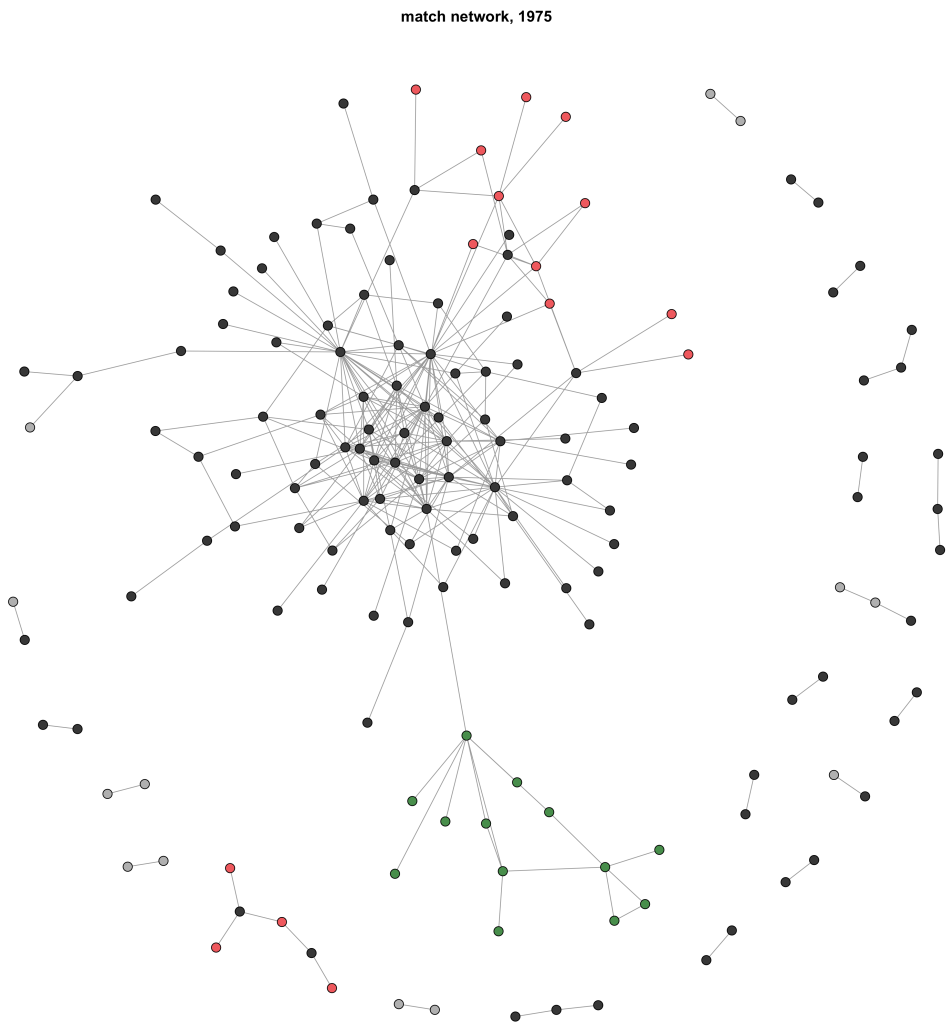

Figure A12: Connections between Go players (points) based on the number of games they played together in the year 1975, organized by a Fruchterman-Reingold layout. Node colors indicate player nationality (Black = Japan, Red = China, Green = South Korea, Blue = Taiwan).

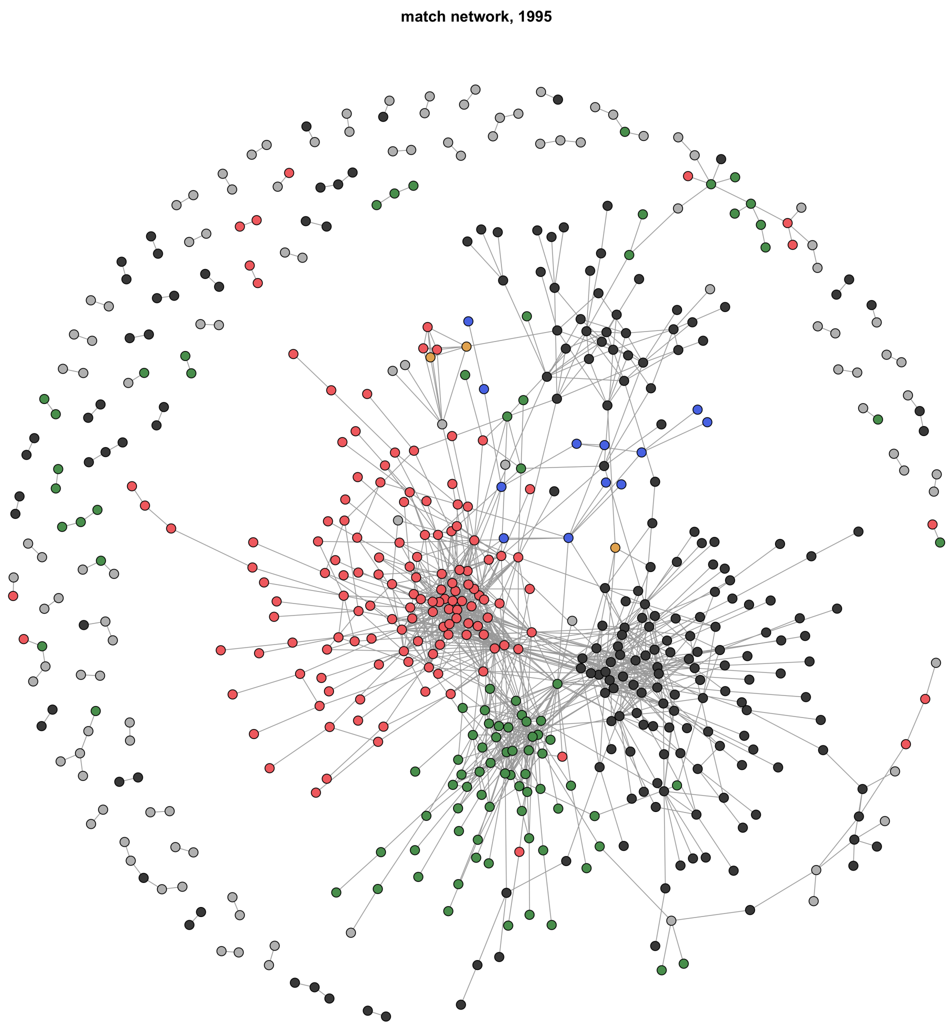

Figure A13: Connections between Go players (points) based on the number of games they played together in the year 1995, organized by a Fruchterman-Reingold layout. Node colors indicate player nationality (Black = Japan, Red = China, Green = South Korea, Blue = Taiwan).

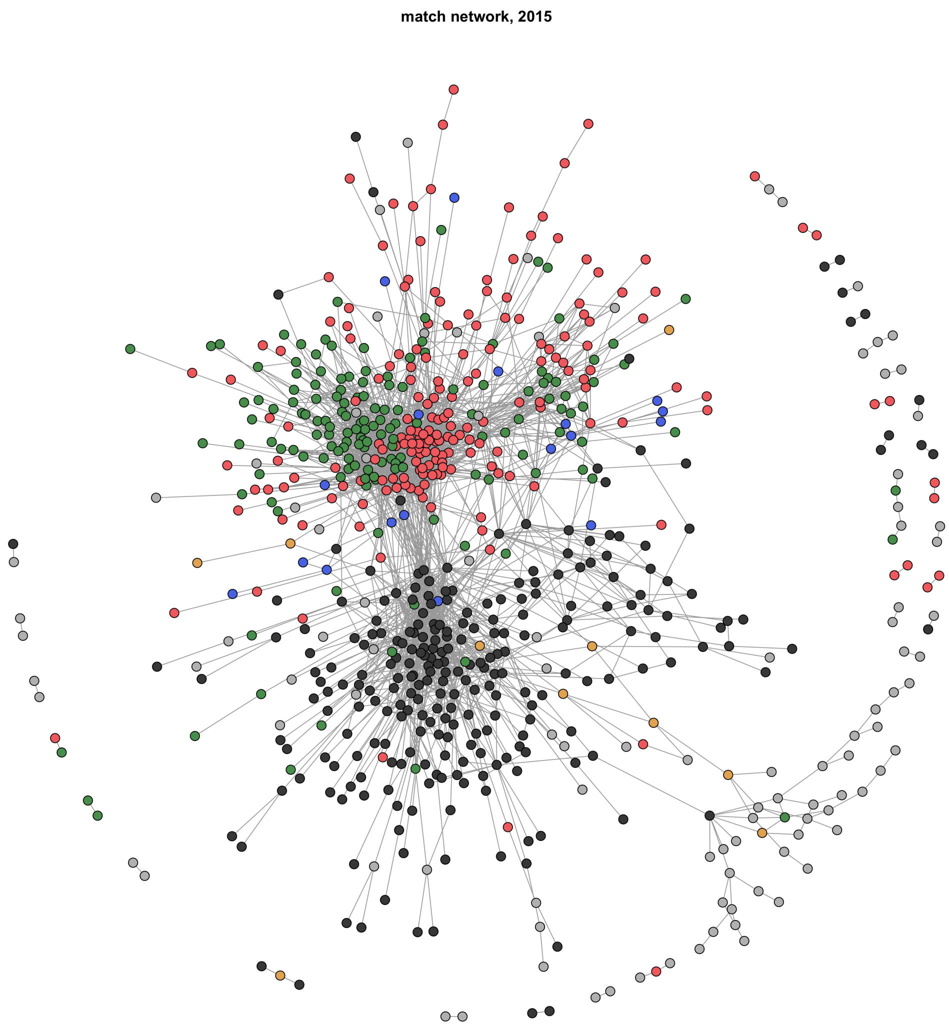

Figure A14: Connections between Go players (points) based on the number of games they played together in the year 2015, organized by a Fruchterman-Reingold layout. Node colors indicate player nationality (Black = Japan, Red = China, Green = South Korea, Blue = Taiwan).

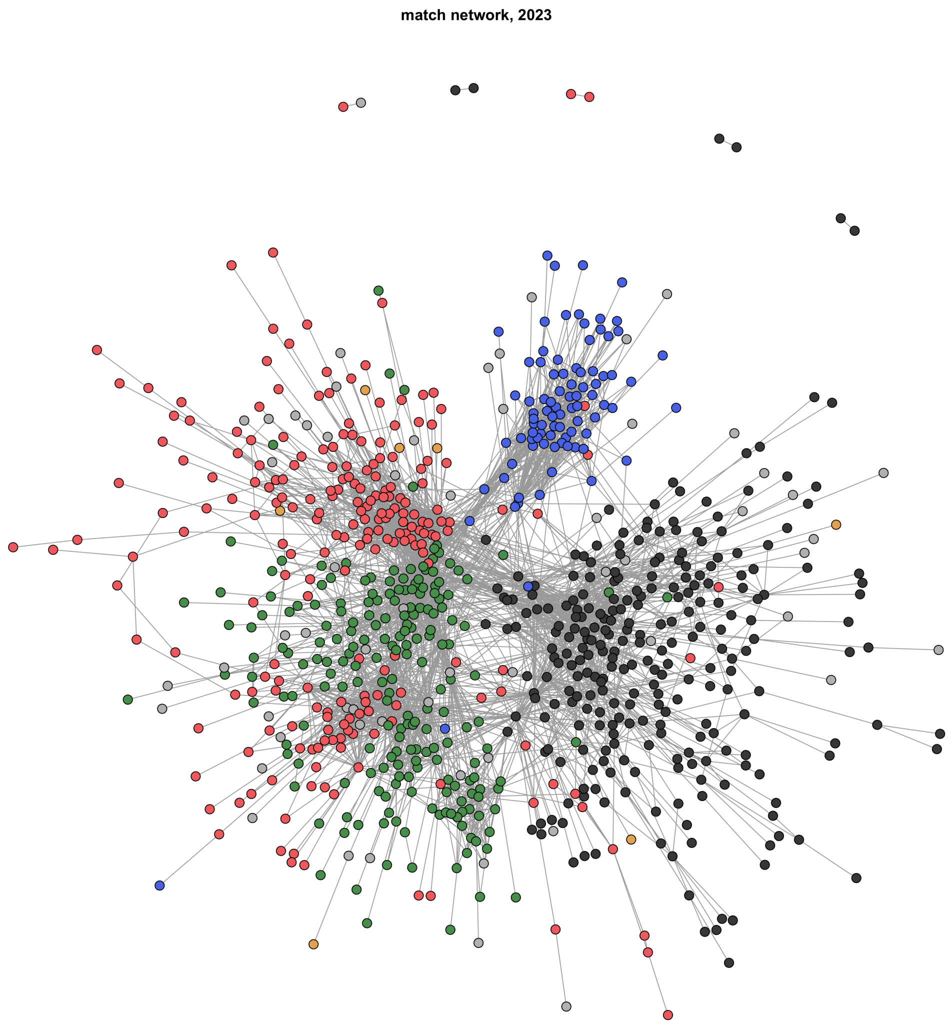

Figure A15: Connections between human Go players (points) based on the number of games they played together in the year 2023, organized by a Fruchterman-Reingold layout. Node colors indicate player nationality (Black = Japan, Red = China, Green = South Korea, Blue = Taiwan).
